# A multi-omic analysis reveals the esophageal dysbiosis as the predominant trait of eosinophilic esophagitis

**DOI:** 10.1186/s12967-023-03898-x

**Published:** 2023-01-25

**Authors:** Luca Massimino, Alberto Barchi, Francesco Vito Mandarino, Salvatore Spanò, Luigi Antonio Lamparelli, Edoardo Vespa, Sandro Passaretti, Laurent Peyrin-Biroulet, Edoardo Vincenzo Savarino, Vipul Jairath, Federica Ungaro, Silvio Danese

**Affiliations:** 1 Department of Gastroenterology and Digestive Endoscopy, IRCCS Ospedale San Raffaele, Milan, Italy; 2 Division of Immunology, Transplantation and Infectious Disease, IRCCS Ospedale San Raffaele, Milan, Italy; 3 IBD Center, IRCCS Humanitas Research Hospital, Milan, Italy; 4 Inserm NGERE, University of Lorraine, Vandoeuvre-les-Nancy, France; 5 Nancy University Hospital, Vandoeuvre-les-Nancy, France; 6 Department of Surgery, Oncology, and Gastroenterology, University of Padua, Padua, Italy; 7 Gastroenterology Unit, Azienda Ospedale Università di Padova, Padua, Italy; 8 Department of Medicine, Division of Gastroenterology, Western University, London, ON Canada; 9 Faculty of Medicine, Università Vita-Salute San Raffaele, Milan, Italy

**Keywords:** Esophagus, Transcriptomics, Web app, Microbiota

## Abstract

**Background:**

Eosinophilic esophagitis (EoE) is a chronic immune-mediated rare disease, characterized by esophageal dysfunctions. It is likely to be primarily activated by food antigens and is classified as a chronic disease for most patients. Therefore, a deeper understanding of the pathogenetic mechanisms underlying EoE is needed to implement and improve therapeutic lines of intervention and ameliorate overall patient wellness.

**Methods:**

RNA-seq data of 18 different studies on EoE, downloaded from NCBI GEO with faster-qdump (https://github.com/ncbi/sra-tools), were batch-corrected and analyzed for transcriptomics and metatranscriptomics profiling as well as biological process functional enrichment. The EoE TaMMA web app was designed with plotly and dash. Tabula Sapiens raw data were downloaded from the UCSC Cell Browser. Esophageal single-cell raw data analysis was performed within the Automated Single-cell Analysis Pipeline. Single-cell data-driven bulk RNA-seq data deconvolution was performed with MuSiC and CIBERSORTx. Multi-omics integration was performed with MOFA.

**Results:**

The EoE TaMMA framework pointed out disease-specific molecular signatures, confirming its reliability in reanalyzing transcriptomic data, and providing new EoE-specific molecular markers including CXCL14, distinguishing EoE from gastroesophageal reflux disorder. EoE TaMMA also revealed microbiota dysbiosis as a predominant characteristic of EoE pathogenesis. Finally, the multi-omics analysis highlighted the presence of defined classes of microbial entities in subsets of patients that may participate in inducing the antigen-mediated response typical of EoE pathogenesis.

**Conclusions:**

Our study showed that the complex EoE molecular network may be unraveled through advanced bioinformatics, integrating different components of the disease process into an omics-based network approach. This may implement EoE management and treatment in the coming years.

**Supplementary Information:**

The online version contains supplementary material available at 10.1186/s12967-023-03898-x.

## Introduction

Eosinophilic esophagitis (EoE) is a chronic inflammatory disease characterized by a T-Helper type 2 (T_H_2) inflammatory response, primarily induced by food antigens, resulting in an accumulation of eosinophils within the esophageal mucosa. The T_H_2-response-specific interleukins were described to play a pivotal role in EoE pathogenesis [1]. To date, EoE is no longer reported as a rare disease since its incidence and prevalence rates are steadily increasing, being 3.7/100,000/year and 22.7/100,000 respectively [2], with a strong male predominance with a 3:1 ratio [3]. Several risk factors are associated with EoE, including allergic/atopic conditions, environmental factors, and lack of *Helicobacter (H). Pylori* infection [4]. However, the genetic predisposition to the disease has been proven, with up to 58% concordance in monozygotic twins [5].

EoE clinical presentation is heterogeneous, with dysphagia and food impaction as the most common symptoms in adults, while heartburn, regurgitation, and feeding intolerance are typical in children. Moreover, a wide range of other symptoms may overlap at any age, such as vomiting, nausea, and chest and/or abdominal pain [1].

At present, endoscopy is the only way to diagnose and monitor the activity of the EoE. Specifically, the diagnosis is defined by a count of > 15 eosinophils per High power field (HPF) at histological evaluation of esophageal biopsies, whereas therapeutic response and remission are defined by a count of ≤ 15 or < 6 eosinophils per HPF, respectively [6]. However, endoscopy remains an invasive diagnostic tool, with low acceptability from the patients and limited availability in clinical practice. With this premise and taking into account the heterogeneity of the clinical onset, the development of new “non-endoscopic” tools aiding diagnostic assessment may be helpful [7].

Therefore, a deeper understanding of the pathogenetic mechanisms underlying EoE remains of paramount importance in order to implement and improve therapeutic lines of intervention and ameliorate overall patient wellness.

Along with the mucosal immunity alterations and fibrosis [8], the epithelial barrier function impairment does remain a hallmark of EoE, in terms of both increased permeability and antigen sensing and presentation roles. Indeed, EoE is featured by a striking pattern of dilated intercellular spaces, with the down-regulation of proteins associated with barrier function and adhesion molecules modulated via an IL-13-dependent mechanism [1]. As a consequence, altered epithelial permeability can lead to a permissive environment that enhances antigen presentation, which in turn leads to persistent chronic inflammation, associated with microbiota dysbiosis [1]. Despite some anatomical and molecular characterizations employed as a kind of gold standard for the definition of EoE pathogenesis, a meta-analysis of the molecular studies may improve the understanding of the physiopathology of the disease and define molecular markers helpful for a straightforward diagnosis of EoE.

We recently developed and released the Inflammatory Bowel Disease (IBD) Transcriptome and Metatranscriptome Meta-Analysis web app (IBD TaMMA, https://ibd-meta-analysis.herokuapp.com/) [9], a complete survey of all public data sets generated for IBD-related studies*.*

Considering the utility that IBD TaMMA has been increasingly showing over the last few months [10] and the continuous access to the platform recorded by Google Analytics (1.8K individual users in October 2022), we here propose a similar meta-analysis of EoE-related public data sets visualized in the EoE TaMMA interactive web app, (https://eoe-meta-analysis.herokuapp.com/; username: ungaro; password: steams).

EoE TaMMA, while confirming well-accepted EoE molecular characteristics, pointed out for the first time that esophageal dysbiosis is the main trait in EoE pathogenesis. Of note, this computational platform may become a precious resource for all clinicians and scientists to expedite discoveries in the field and ameliorate the overall understanding of EoE pathophysiology, which urgently needs further implementation.

## Materials and methods

All authors had access to the study data and reviewed and approved the final manuscript.

### Transcriptomics analysis

**Fig. 1:**
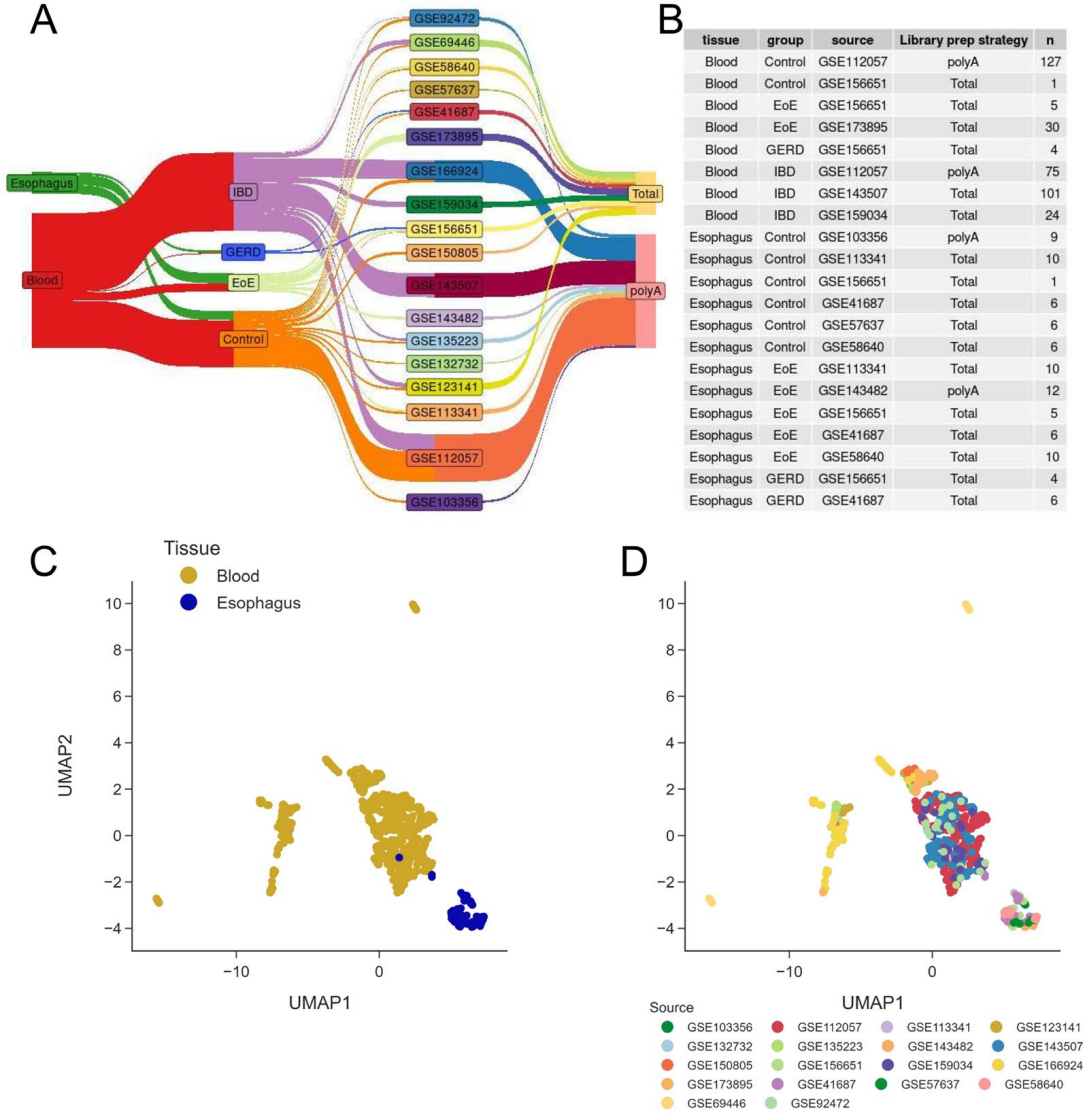
EoE TaMMA overview. **A** Sankey plot showing the relationships between different metadata. **B** Table listing the details of the studies analyzed within the EoE TaMMA web app. **C**, **D** Sample distribution by multidimensional scaling of the human whole transcriptome by UMAP from patients with EoE, GERD, IBD, and healthy controls, where the closer the samples are, the higher the similarity between their transcriptomes

RNA-seq data were downloaded from NCBI GEO (18 different studies, listed within the EoE TaMMA web app, in the metadata tab > analysis overview subtab, and metadata tab > sample characteristics subtab; Fig. 1A, B, and Additional file 2: Table S1) with faster-qdump (https://github.com/ncbi/sra-tools). FASTQ sequencing reads were adaptor-trimmed and quality-filtered with Trimmomatic [11], prior to mapping to the hg38 human reference genome with STAR [12]. Gene count normalization and differential gene expression were performed with DESeq2 [13]. Functional enrichment analysis was done with GeneSCF [14]. Low-dimensional embedding of high-dimensional data was performed by either Uniform Manifold Approximation and Projection (UMAP) or t-distributed stochastic neighbor embedding (t-SNE) machine learning algorithms, within R (https://cran.r-project.org/web/packages/umap).

Statistical significance was set at FDR < 1e−5.

### Web app design

The web app, available at https://eoe-meta-analysis.herokuapp.com/ (username: ungaro; password: steams), was designed with plotly and dash (https://plotly.com/dash/). Processed data and code are hosted by GitHub and are available at https://github.com/DU-omics/eoe-meta-analysis_data and https://github.com/DU-omics/eoe-meta-analysis. The complete guide and the related documentation are available at https://ibd-tamma.readthedocs.io/. The user interface is described in Additional file 1: Fig. S1.

Newly released EoE datasets will be continuously searched and implemented in this platform to maintain it as timely updated. A suggestion link is available at the bottom of the home page of the platform for the users to notify newly released data sets.

### Meta-analysis

Batch-effect detection and correction were performed as previously described [9, 15], in accordance with source (batch covariate) and tissue of origin (explaining other possible covariance), with ComBat [16], within the Surrogate Variable Analysis v1.8 R package (https://bioconductor.org/packages/release/bioc/html/sva.html).

### Metatranscriptomics analysis

Metatranscriptomics was performed as previously described [9, 15]. The reads that failed to align to the human genome were subsequently mapped to the complete collection of all available microbial genomes (https://www.ncbi.nlm.nih.gov/genome) with Kraken2 for exact alignment of k-mers and accurate viral read classification [17]. Relative abundances and differential analysis was performed with DESeq2 upon variance-stabilizing transformation [13]. Microbial read calls were confirmed by manually aligning Kraken2-classified reads to the respective viral genomes with Bowtie2, and visualizing the resulting BAM alignments with the Integrative Genomics Viewer (IGV) [18]. Before statistical analysis, classified reads were double-checked with FastQC (https://www.bioinformatics.babraham.ac.uk/projects/fastqc) to confirm quality filtering and adaptor trimming and then submitted to BLAST [19] to exclude possible artifacts resulting from the in silico analysis. Species alpha diversity and dominance indices were calculated with vegan (https://cran.r-project.org/web/packages/vegan). The statistical significance threshold was set at P < 0.05.

### Single-cell RNA-seq data analysis and deconvolution

Tabula Sapiens raw data [20] were downloaded from the UCSC Cell Browser (https://cells.ucsc.edu). Esophageal single-cell raw data were downloaded from https://www.ncbi.nlm.nih.gov/geo/query/acc.cgi?acc=GSE175930 [21]. The analysis was performed within the Automated Single-cell Analysis Pipeline (ASAP) [22]. Single-cell data-driven bulk RNA-seq data deconvolution was performed with MuSiC and CIBERSORTx [23, 24].

### Multi-omics factor analysis

The different omics datasets were integrated with the Multi-Omics Factor Analysis (MOFA) framework [25] which interprets multi-layer (different data modalities) high-dimensional data and infers an interpretable low-dimensional representation in terms of a few latent factors. Variance stabilization and z-scoring, followed by feature selection to select the most informative variables, namely those explaining more variance, were performed to ensure that all the molecular layers were equally represented. Variance decomposition was then performed between groups to find differences in terms of variance explained within factors and groups, thus stratifying patients into cohorts, each one of them displaying a specific molecular signature.

## Results

### The EoE TaMMA web app identifies EoE-specific markers

EoE aetiopathogenesis is not fully explained, even if a major shift toward antigen-mediated T_H_2 response has been accepted as the most relevant characteristic [1]. Although some RNA-seq studies have been performed, the complete survey of all transcriptomics collections to advance EoE-related research has not been compiled yet. For this purpose, we analyzed and batch-corrected a total of 18 different studies, including 660 samples from esophageal mucosa and blood, combined into the EoE TaMMA web app (Fig. 1A and B).

**Fig. 2:**
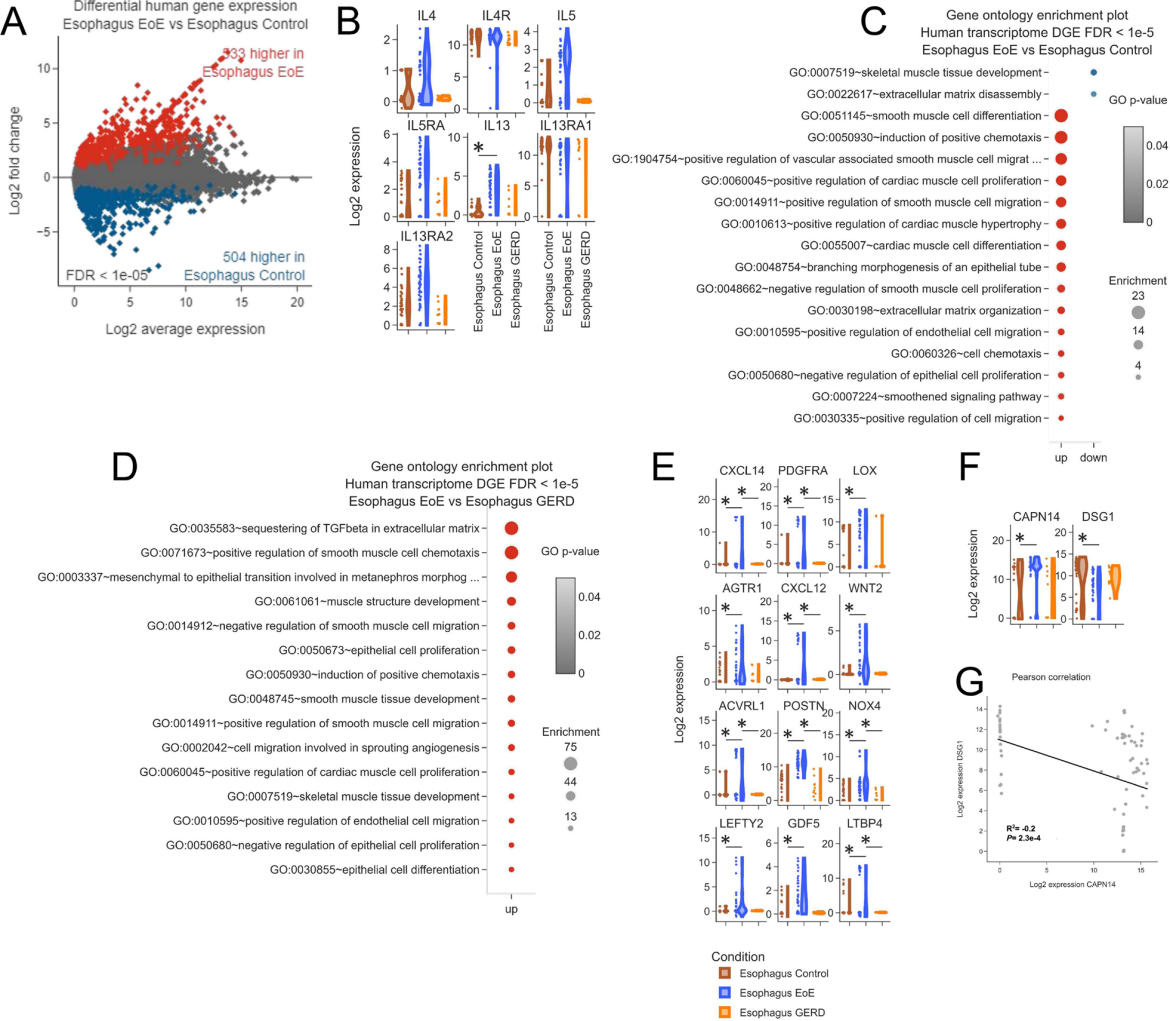
EoE TaMMA confirms EoE-specific traits. **A** MA plots showing the differential gene expression results expressed as log2(fold change) in the indicated comparisons as a function of log2(average gene expression). Red dots represent genes being differentially expressed with high statistical significance (false discovery rate (FDR) < 1 × 10^−5^). The number of differentially expressed genes and their trends are indicated in red and blue for the up and down-regulated genes, respectively. **B** Violin plots showing differential normalized expression of the indicated genes among EoE, GERD, and control esophagi. **C**, **D** GO plot showing modulation of biological processes related to epithelial cell proliferation, smooth muscle cell migration, proliferation, differentiation, extracellular matrix remodeling, and chemotaxis between EoE and control (**C**) and EoE and GERD (**D**). **E**, **F** Violin plots showing differential normalized expression of the indicated genes among EoE, GERD, and control esophagi. The asterisks indicate FDR < 1 × 10^−5^.** G** Pearson correlation analysis between CAPN14 and DSG1 expression levels expressed as log2(fold change)

We included blood and esophageal tissues from EoE and GERD patients and IBD-derived blood samples. Of note, we included also IBD samples because it helped to better correct the batch variability, a normal consequence of the combination of different studies coming from a variety of data sources generated by different operators, sequencers, and analytic platforms [9, 10]. However, IBD characterization was not shown but can be fully browsed at the dedicated platform (IBD TaMMA). After batch correction, esophagus and blood-derived samples appeared as two distinct clusters (Fig. 1C), despite the different study sources (Fig. 1D), indicating that the correction approach was effective in rendering samples harmonized and comparable. Differential gene expression (DGE) analysis revealed 533 and 504 genes up- and down-regulated, respectively, in the EoE esophagus by comparison with the control (Fig. 2A).

Since the role of T_H_2 cytokines is key in EoE pathogenesis, we specifically evaluated the expression of interleukin (IL)13, IL4, IL5, and their receptors [26]. According to a previously published EoE single-cell (sc)RNA-seq [21], IL13 and IL5 were broadly expressed by pathogenic effector GATA-3 T_H_2 cells, expanded in EoE tissue biopsies [21] (Additional file 1: Fig. S2A, and Additional file 1: Fig. S3M and 3P). IL4 was expressed by Treg exclusively (Additional file 1: Fig. S3D and 3P), while IL4 receptor by both the stromal and immune compartments (Additional file 1: Fig. 3E, J, M–O). Additionally, IL5RA resulted as expressed by all myeloid cells, among which CLC-expressing eosinophils (Additional file 1: Fig. 3F, N, O).

**Fig. 3:**
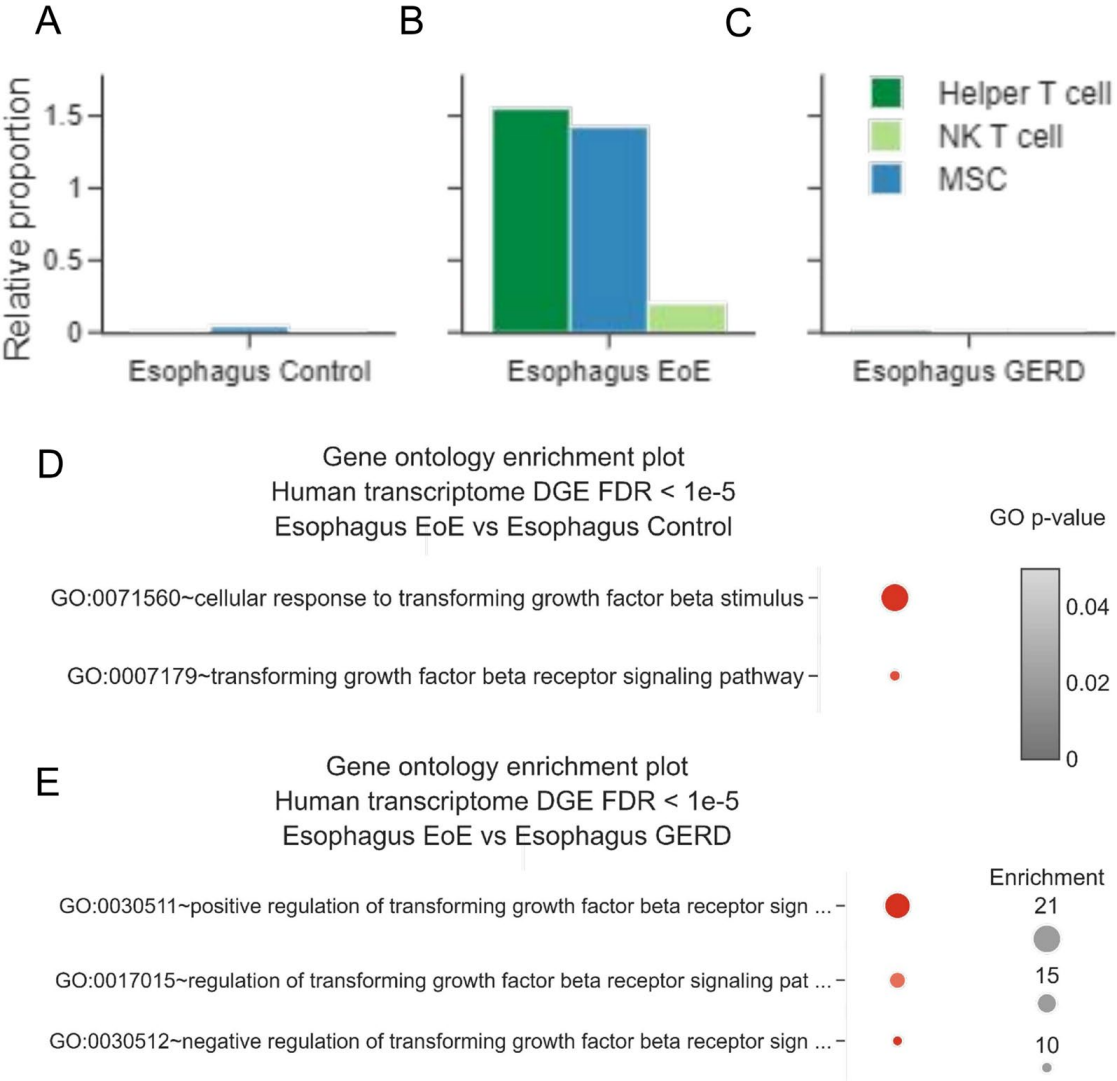
Computational deconvolution of EoE-TaMMA bulk transcriptomic. **A**, **B** Bar plots showing the differential proportion of the indicated cell populations in Control (**A**), EoE (**B**), and GERD (**C**). **D**, **E** GO plots showing modulation of biological processes related to the transforming growth factor beta between EoE and control (**D**) and EoE and GERD (**E**)

In EoE TaMMA, IL13 was the sole confirmed as upregulated in the EoE esophagus as compared to the control, while IL5, IL4, and IL13, IL4, and IL5 receptors were not significantly modulated, although a trend was observed (Fig. 2B). Additionally, IL13 did not result in a specific trait of EoE when compared with GERD-derived samples (Fig. 2B and Additional file 1: Fig. 4A). This evidence might support the difficulties in a straightforward diagnosis for patients with EoE and GERD-shared symptoms [1].

**Fig. 4:**
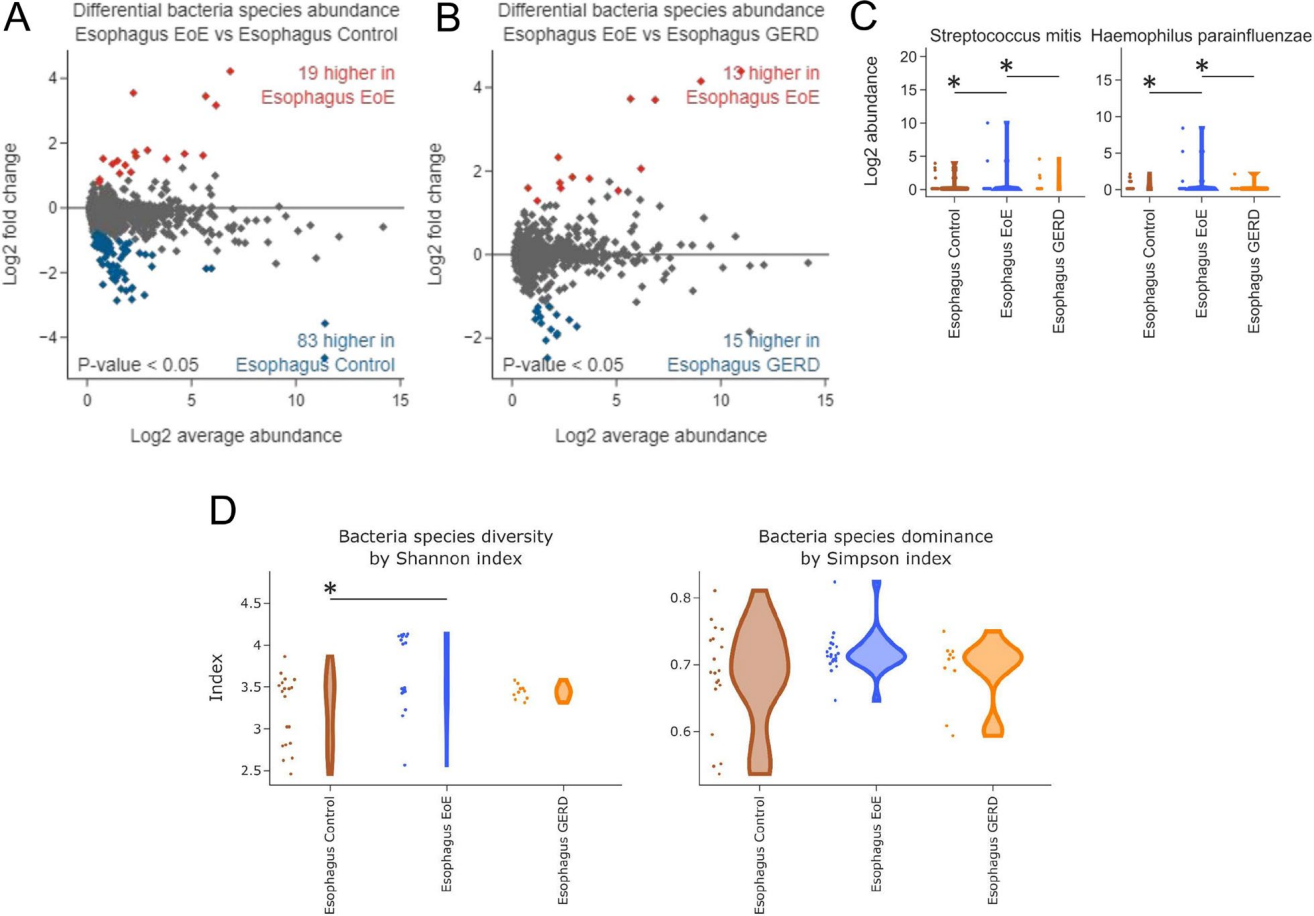
**A**, **B** MA plots showing the differential abundances expressed as log2(fold change) between the indicated comparisons. Red dots represent bacterial species being differentially expressed with high statistical significance (P < 0.05). The number of differentially expressed genes and their trends are indicated in red and blue for the up and down-regulated genes, respectively. **C** Violin plots showing differential normalized expression (log2 fold change) of the indicated bacterial species among EoE, GERD, and control esophagi. The asterisks indicate P < 0.05. **D** Violin plots showing the Shannon and Simpson indices among EoE, GERD, and healthy esophagi. The asterisks indicate P < 0.05

Nonetheless, we sought to further characterize and confirm EoE-related traits in our TaMMA platform. IL13 is known as a mediator of a series of processes in allergic diseases, such as eosinophil chemotaxis, epithelial (goblet) cell proliferation, collagen deposition, and smooth muscle contractility [26], thus prompting us to evaluate these features in EoE esophagi. Therefore, by gene ontology (GO) analysis, we observed biological processes related to epithelial cell proliferation, smooth muscle cell migration, proliferation, differentiation, extracellular matrix remodeling, and chemotaxis to be modulated in EoE by comparison with the control tissues (Fig. 2C).

Interestingly, we also found these biological signatures to be modulated when EoE tissues were compared to the GERD (Fig. 2D), indicating that the EoE pathogenesis is different from the GERD concerning these aspects.

We then evaluated which genes were involved in these biological process alterations and distinguished EoE from GERD in terms of expression levels. Besides the already known factor CCL26 (Eotaxin-3) expressed by stromal and epithelial cells (Additional file 1: Fig. S3G, 3J and 3L) known to regulate the eosinophilic trafficking to the esophagus in patients with EoE and to discriminate between EoE and GERD [27], other markers were pointed out, such as CXCL14, PDGFRA, CXCL12, ACVRL1, POSTN, NOX4 and LTBP4 (Fig. 2E). These results provided evidence that a composite panel of markers specific to EoE may be developed to make the diagnosis more accurate.

Furthermore, IL13 was acknowledged as a factor that induces calpain 14 (CAPN14) expression (Additional file 1: Fig. S3H and 3J), which affects the epithelial barrier through the degradation of the desmosomal protein desmoglein 1 (DSG1) [28]. Our analysis revealed increased CAPN14 expression in the EoE esophagus by comparison with the control, while DSG1 was found down-regulated. (Fig. 2F, G), supporting the inverse relationship existing between these two proteins in the epithelial barrier [28]. Even if the bulk sequencing data are key for understanding the molecular process in a biological system, the great limitation remains the unavailability of information regarding the proportion of cell types within a sample. Nonetheless, in recent decades, approaches like computational deconvolution of single-cell RNA-seq data have been developed and optimized to obtain such information starting from whole tissue expression profiling data [29]. Deconvolution is a time and cost-efficient approach for obtaining cell type-specific information from bulk gene expression of heterogeneous tissues, providing an estimation of cell-type proportions or abundances in samples.

To this end, we exploited *Tabula Sapiens*, a multiple-organ, single-cell transcriptomic atlas of human tissues [30]. The analysis performed on the EoE TaMMA data confirmed the increased proportion of T helper cells, described as part of the EoE pathogenic process [31], by comparison with both the healthy and GERD tissues.

Furthermore, considering the role of invariant (i)NKT cells, also known as classical NKT cells, during EoE pathogenesis [32], we verified and confirmed their increased proportion specifically in EoE tissues (Additional file 1: Fig. S5A–F).

Tissue remodeling by increased collagen deposition, matrix disassembly, and epithelial-to-mesenchymal (EMT) transition are phenomena that lead to the peculiar fibrostenotic aspect of an EoE esophagus [33]. During fibrotic complications, epithelia lose many characteristics, such as polarity, specific markers, and tight junctions, and acquire properties of mesenchymal cells, including motility, loose cell adhesion via N-cadherin, and de-polarized cytoskeletal arrangements such as vimentin [34]. Consistently, we observed an increased proportion of mesenchymal stem cells (MSC) in EoE compared to GERD and healthy samples (Fig. 3A–C), thus confirming the pro-fibrotic status of the esophagus in EoE conditions. This finding may have implications for developing prognostic molecular markers predicting the risk of fibrostenosis in EoE patients.

These data were also paralleled by the dysregulation of biological processes related to the transforming growth factor beta (TGFB), which was found to increase in the EoE by comparison with both the healthy and the GERD tissues (Fig. 3D and E), with specific markers distinguishing between EoE and GERD (Fig. 2E, specifically: WNT2, ACVRL1, POSTN, NOX4, LEFTY2, GDF5, and LTBP4), supporting the notion that the tissue remodeling and fibrotic process are associated with EoE pathogenesis [26].

Overall, these results pinpointed the EoE TaMMA web app as a reliable tool, evidencing the main hallmarks of EoE, often different from GERD, and thus resulting in a powerful asset for expediting research with novel insights into both pathogenesis and approaches for a more accurate diagnosis of EoE.

### EoE TaMMA reveals microbiota dysbiosis as a predominant characteristic during EoE pathogenesis

As mentioned above, EoE TaMMA provides a wide picture of omics profiling of EoE tissues, not only confirming the already known molecular landscape associated with EoE but also pointing out new insights for further investigation of their complex pathogenesis. For instance, among all the markers that were pointed out as specifically determining EoE (Fig. 2 and Additional file 1: Fig. S1), CXCL14, a chemoattractant chemokine expressed by the epithelium, stromal cells and by monocytes (Additional file 1: Fig. S3I, 3J, 3L, and 3R), gained our attention because of its documented antimicrobial activities against pathogens [35] and its higher level in EoE compared with both control and GERD, suggesting possible EoE-specific microbial signatures different from the GERD.

Thus, going deeper into the microbiota profiling, EoE TaMMA pointed out the bacterial species as the most differentially dysregulated microbial entities among EoE, GERD, and control esophageal tissues (Fig. 4A and B and Additional file 1: Fig. S6A–6F).

We then intersected the bacterial species highly abundant in EoE by comparison with the healthy or GERD and identified the 9 candidates specifically characterizing the EoE esophagus (Additional file 1: Fig. S6G). The most abundant were the *Streptococcus mitis* and *Hemophilus parainfluenzae* (Fig. 4C)*,* normally colonizing the oropharynx tract and already reported as being associated with EoE pathogenesis [36]. Moreover, increased bacterial diversity but no species dominance was found in EoE esophagi as compared to controls (Fig. 4D), despite previous studies reporting no differences between these experimental groups [7, 36–38]. Such a discrepancy might be explained by the former small-sized samples and the consequent lower statistical power that might have contributed to the loss of significant signals that, by contrast, our analysis pointed out.

This is an example of how this computational approach may be exploited to highlight specific signatures. Nevertheless, dissecting each omic at a time would be a huge effort, and many statistically relevant details could be lost.

Multi-omics approaches, often supported by machine-learning algorithms [39], are facilitating the discovery of new molecular networks and hubs by comprehensively and simultaneously analyzing different data layers, such as the human transcriptome and metatranscriptome [40]. Also, it can allow the identification of the origin of patient heterogeneity, ultimately stratifying them based on their molecular characteristics. Indeed, this methodological approach mitigates intersubject variability thanks to the discovery of the principal sources of variation in multi-omics data sets. In this regard, the possibility to perform such an analysis recently became effective thanks to the machine learning-based tool Multi-Omics Factor Analysis (MOFA). MOFA infers a set of (hidden) factors that capture biological and technical sources of variability [25].

**Fig. 5:**
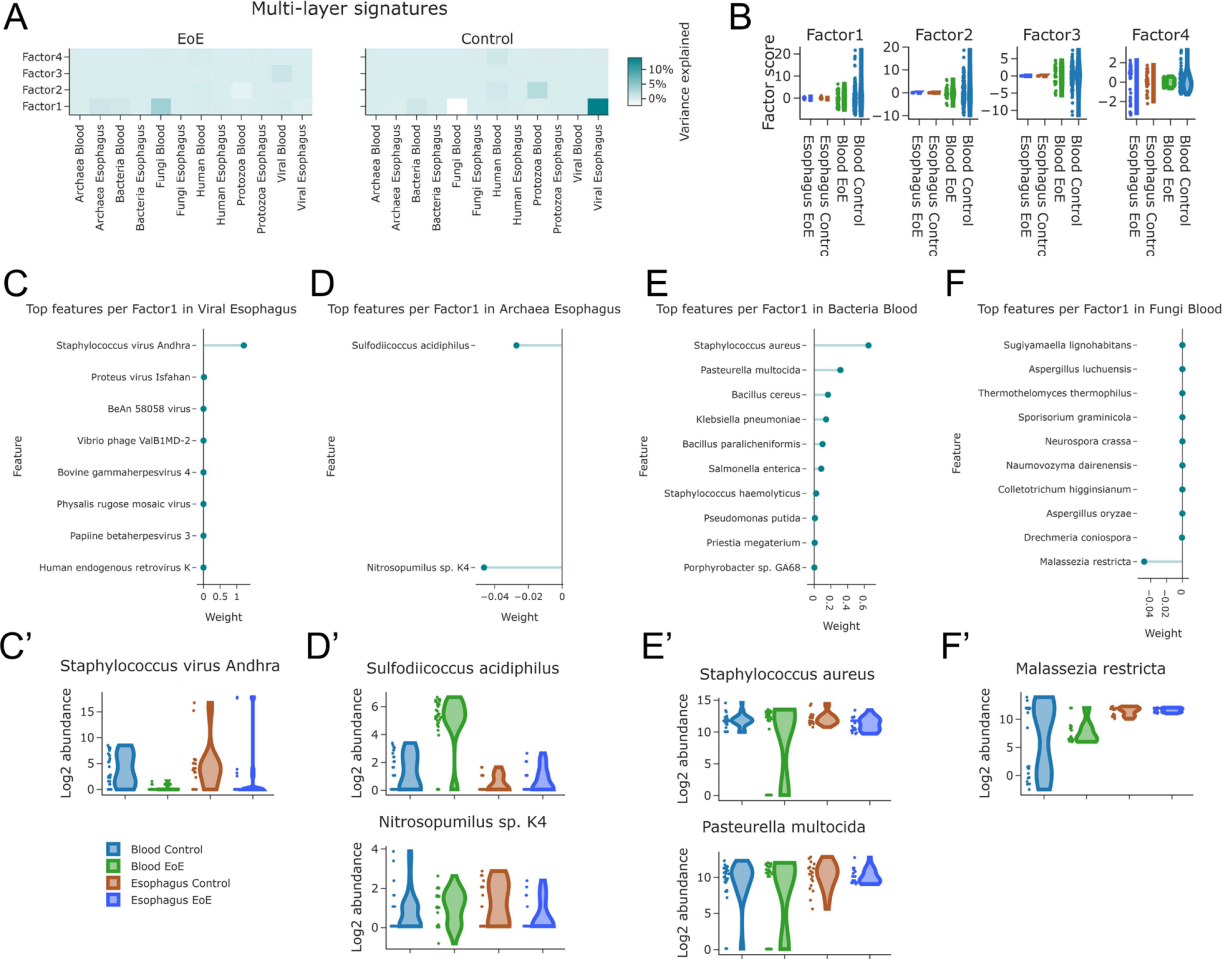
Multi-omic analysis in EoE TaMMA. **A** Heatmaps showing the omics categories explaining the highest amount of variance for each factor found by MOFA in EoE and control. **B** Violin plots showing composite molecular signature scores within conditions. **C**–**F** Needle plots showing weights representing the variance explained by each feature for the indicated factors and layers (**C**–**F**) and violin plots showing the relative abundance of top features within conditions (**C**′–**F**′)

Therefore, by applying MOFA for processing the six different types of omics data, encompassing the human transcriptome, virome, eukaryome (fungi and protists), bacteriome, and archaeome from EoE and healthy control samples (Additional file 1: Fig. S7A), the source of variation between the EoE and healthy (control) esophageal mucosa was identified mainly among the metatranscriptomics (microbiome) factors. Specifically, a subset of archaea, fungi, protozoa, and viral species and, to a lesser extent some human transcripts, allowed the development of 4 multi-layers molecular signatures able to distinguish EoE patients from controls (Fig. 5A), indicating that microbial dysbiosis may be a key player during the EoE pathogenesis.

Going deeper into the analysis, the primary source of variance between EoE and control was found at the level of factor 1 mainly in the esophageal virome and archaeon composition and in the blood bacteriome and mycome (fungi, Fig. 5B–F). The top features within factor 1, showing a high impact (weight) in explaining the variance, were the *Staphylococcus virus Andhra* (Fig. 5C and C′), the *Sulfodiicoccus acidophilus,* and the *Nitrosopumilus *sp*. K4* (Fig. 5D and D′), the *Staphylococcus aureus* and the *Pasteurella multocida* (Fig. 5E and E′)*,* and the *Malassezia restricta* (Fig. 5F and F′). Besides the factor 1-driven stratum of patients, the factor 2-driven defined another subset of human subjects with EoE where the *Plasmodium knowlesi* explained the majority of the variance in the protozoa profiling of the blood (Additional file 1: Fig. S7B and 7B′), while factor 3 was featured by the *Proteus virus Isfahan,* explaining most of the variance in this stratum of patients (Additional file 1: Fig. S7C and 7C′).

**Fig. 6:**
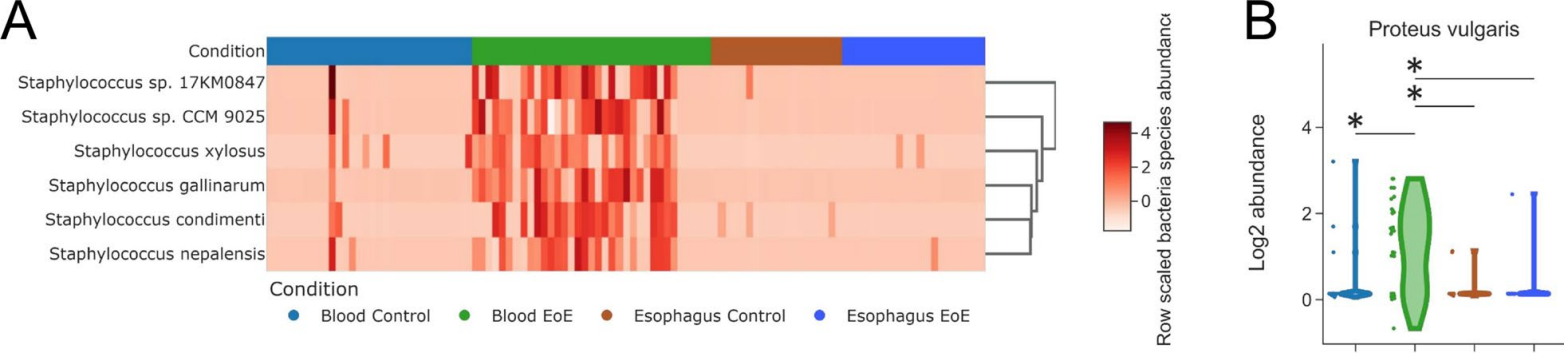
EoE TaMMA reveals specific microbiota composition in EoE. **A** Heatmap showing the different Staphylococcus species colonizing EoE and control esophagi and blood. **B** Violin plots showing the differential normalized abundance of the Proteus vulgaris among EoE, and control esophagi and blood. The asterisks indicate P < 0.05

Of note, since Staphylococcus virus Andhra parasitizes Staphylococci we checked the levels of these bacteria, but no differences in the esophagi were found (Fig. 6A).

Regarding the *Proteus* species (i.e., *mirabilis*), they are known to be parasitized by the Proteus virus Isfahan [41]. Thus, we wondered whether some *Proteus* species could change their levels according to the Proteus virus Isfahan abundance. Interestingly, *Proteus vulgaris* was pointed out as highly abundant in EoE blood by comparison with the control, while no differences in the esophagus were found (Fig. 6B).

Based on these pieces of evidence, we can speculate that the presence of defined classes of microbial entities in specific subsets of patients may participate in inducing the antigen-mediated response typical of EoE pathogenesis.

## Discussion

EoE is a complex, clinically heterogeneous disease where many factors have been proposed to interact with each other and lead to chronically inflamed esophageal mucosa, with upper gastrointestinal symptoms that range from dysphagia to esophageal food impaction [42].

Even if some treatments are available, EoE remains a chronic disease, compromising the overall patients’ quality of life. Thus, having more mechanistic details of EoE pathogenesis may enable and support the development of new therapeutic lines of intervention. Moreover, molecular profiling may pave the road for further clinical phenotyping of EoE, ultimately reflecting better-personalized care.

In recent years, the analysis of different molecular aspects in the same patients with complex pathologies has often become one of the most powerful scientific approaches that have led, in some cases, to the discovery of disease-combined characteristics that remained hidden for a long time [43].

For this purpose, we recently released the IBD TaMMA framework [9], which is currently exploited worldwide as a support for research and has already led to new scientific outputs further dissecting the complexity and heterogeneity of IBD pathogenesis [9, 41, 44]. Hence, we sought to create a similar computational framework for EoE by surveying the already published transcriptomics studies. The EoE TaMMA framework confirmed some well-accepted EoE traits and can therefore be considered a useful and reliable web app and a resource for fostering novel fields of research. EoE TaMMA confirmed IL13 upregulation in the EoE esophagus by comparison with the control, while IL5, IL4, and the IL13, IL4, and IL5 receptors were not significantly modulated. IL13 was associated with EoE pathogenesis from a clinical standpoint [25, 26], and the IL13 signaling inhibition was described as successful in the treatment of this disorder [45]. In this regard, we can speculate that Dupilumab, a monoclonal antibody against the IL4 receptor, mediating both IL4 and IL13 pathways, was found effective in phase 2 randomized trial of EoE patients [45] by interfering with the IL13, rather than the IL4 signaling, justifying the absence of statistical significance in the IL4 modulation in our platform. Further clarifications will come from other transcriptomics studies with larger sample sizes that, once they will be available to the scientific community, will be integrated within the web app to better explain the role of IL4 and IL5 in EoE pathogenesis. Indeed, despite some pieces of evidence in experimental models of EoE describing IL4 and IL5 as possible actors in EoE [25, 46–48], the direct link between the pathogenesis and their specific roles has not been uncovered yet [49]. This may explain the lack of statistical significance in the differential expression of these two factors between EoE and control esophagi that certainly need further investigations and demonstration in future experimental models of EoE.

After pursuing an optimal batch correction, EoE TaMMA helped to draw molecular and biological signatures able to distinguish EoE from GERD, often sharing symptomatic esophageal patterns. Therefore, such a specific profile, including increased levels of CXCL14, PDGFRA, CXCL12, ACVRL1, POSTN, NOX4, and LTBP4 in EoE by comparison with the GERD and control samples, may guide the correctness of diagnosis and might help to design accurate diagnostic panels. Future studies will clarify whether this may help the identification of clinically relevant phenotypes of EoE (refractory or aggressive fibrostenotic forms), which may benefit from personalized therapeutic approaches.

Although the microbiota has been already considered a player in EoE pathogenesis, so far no indication regarding its high impact on EoE pathogenesis was provided. Our MOFA-driven multi-omics revealed that the 4 factor-based patient stratification was mainly characterized by differential microbiota compositions. Besides the well-known bacterial dysbiosis, the other microbial components (archaea, fungi, viruses, protozoa) were unveiled to be part of patient microbiota compositions that might drive patient stratification in future studies on EoE-affected cohorts.

The results obtained indicated two main pieces of evidence: (i) MOFA is useful for characterizing the source of diversity in patients with gastrointestinal diseases; (ii) patients can be stratified by MOFA-identified factors defining sub-cohort of patients displaying stratum-specific molecular signatures, that we sought to explain. For example, the *Staphylococcus virus Andhra* was shown to act as an antimicrobial commensal by inhibiting the growth and degrading the cell walls of diverse *Staphylococci *[50]. Its abundance, higher in the control than in the EoE (Fig. 5C) might be consistent with its protective function against detrimental commensals, such as other *Staphylococci found* highly abundant in the EoE blood by comparison with the control. Interestingly, in the esophagi no differences in these bacterial species levels were found (Fig. 6A), supporting the indication of the EoE as a systemic rather than a local disease [51].

Similarly, the *Proteus virus Isfahan* acts as a lytic *Proteus* phage active against planktonic and biofilms of *Proteus mirabilis* [52]. *Proteus* species, low-abundance commensals of the human gut, possess many virulence factors that have been recently proposed as relevant to gastrointestinal disease pathogenesis and associated with alterations of gut motility and adherence [53]. Notably, from our analysis, *Proteus vulgaris* was pointed out as highly abundant in EoE blood by comparison with the control, while no differences in the esophagus were found (Fig. 6B) These results suggested that blood virome dysbiosis might enhance the expansion of pathogenic bacteria that in turn promote EoE pathogenesis in a specific cluster of patients.

*Sulfodiicoccus acidophilus* and *Nitrosopumilus *sp*. K4* are thermoacidophilic and ammonia-oxidizer archaea, respectively, poorly studied in the context of human disease pathogenesis. In recent years, the archaeome has been acquiring a great interest in the context of chronic intestinal inflammation and its dysbiosis has been reported to modulate mucosal homeostasis [54]. Therefore, the investigation of archaeal dysbiosis associated with esophagitis is worthwhile. As reported in Fig. 5D′, the *Sulfodiicoccus acidophilus* and the *Nitrosopumilus *sp*. K4* are increased and decreased, respectively, in EoE compared to the healthy samples. Their differential abundances may indicate their potential roles in EoE pathogenesis, despite the current lack of knowledge in the field. Further studies may better characterize the possible mechanisms driven by these archaeal species during the EoE pathogenesis.

The *Staphylococcus aureus,* and the *Malassezia restricta*, although not significantly modulated between EoE and healthy controls (Fig. 5E and F), may underlie the pathogenesis in specific cohorts of patients (Factor-1 driven patient stratum) by stimulating the allergic response through the release of antigenic proteins [55, 56], while the *Pasteurella multocida* may manipulate T cell differentiation through the release of specific toxins [57].

Similarly, we do not exclude that *Plasmodium knowlesi, which* emerged from our analysis as a specific feature of the factor 2-driven patient stratum, may act as a microbial commensal stimulating the allergic response in these patients, despite its classification as a zoonotic malaria parasite [58].

One major limitation of this meta-analysis at the moment is the intrinsic lack of other information, such as clinical metadata. Indeed, the transcriptomic studies included in the framework missed, for example, information on the treatment types, localization of the disease, if nonerosive esophageal reflux or GERD, or patients’ age, as well as many other important characteristics that, whether annotated, could have helped to assign to a cluster of patients specific clinical characteristics. Future implementation of the framework including this information may allow the patient clustering, virtually addressing them to tailored treatments.

Another intrinsic limitation of the platform is the small number of studies profiling the EoE samples if compared to those included in the IBD TaMMA, so we encourage scientists to perform transcriptomics experiments that will enable the platform to achieve much higher statistical power.

Despite these limitations, we believe the web app is helpful for other scientists who may use the EoE TaMMA-described features to foster new hypotheses and concepts for developing more accurate and personalized therapies.

## Conclusions

Our study represents a step forward to possibly unravel patient heterogeneity through advanced bioinformatics, integrating different components of the disease process into an omics-based network approach that sought to unravel the molecular landscape of EoE patients and to solve its intricacy, with a promise of better patient management and treatment in a short-term future.

## Supplementary Information

**Additional file 1.** Additional Figures.

**Additional file 2.** Additional Table.

## Data Availability

Transcriptomic data are accessible at https://eoe-meta-analysis.herokuapp.com/. Processed data and code are available at https://github.com/DU-omics/eoe-meta-analysis_data/master/, and https://github.com/DU-omics/eoe-meta-analysis, respectively. Data, analytic methods, and study materials will be made available upon request to the authors.

## Abbreviations

EoE: Eosinophilic esophagitis
TaMMA: Transcriptome and Metatranscriptome Meta-analysis
GERD: Gastroesophageal reflux disorder
T_H_2: T-Helper Type 2
HPF: High power field
IBD: Inflammatory Bowel Disease
IL: Interleukin
ILR: Interleukin receptor
sc: Single-cell
RNA-seq: RNA sequencing
CPN14: Calpain 14
DSG1: Desmosomal protein desmoglein 1
CXCL: C-X-C motif ligand
EMT: Epithelial-to-mesenchymal transition
TGFB: Transforming growth factor beta
MOFA: Multi-Omics Factor Analysis
