# Supplementary material for: A multi-omic analysis reveals the esophageal dysbiosis as the predominant trait of eosinophilic esophagitis
Source: J Transl Med. 2023 Jan 25;21:46. doi: 10.1186/s12967-023-03898-x (PMC9875471; doi:10.1186/s12967-023-03898-x)
Supplement: Supplementary file 1 — Additional file 1. Additional Figures. [file 12967_2023_3898_MOESM1_ESM.pdf]

Figure S1

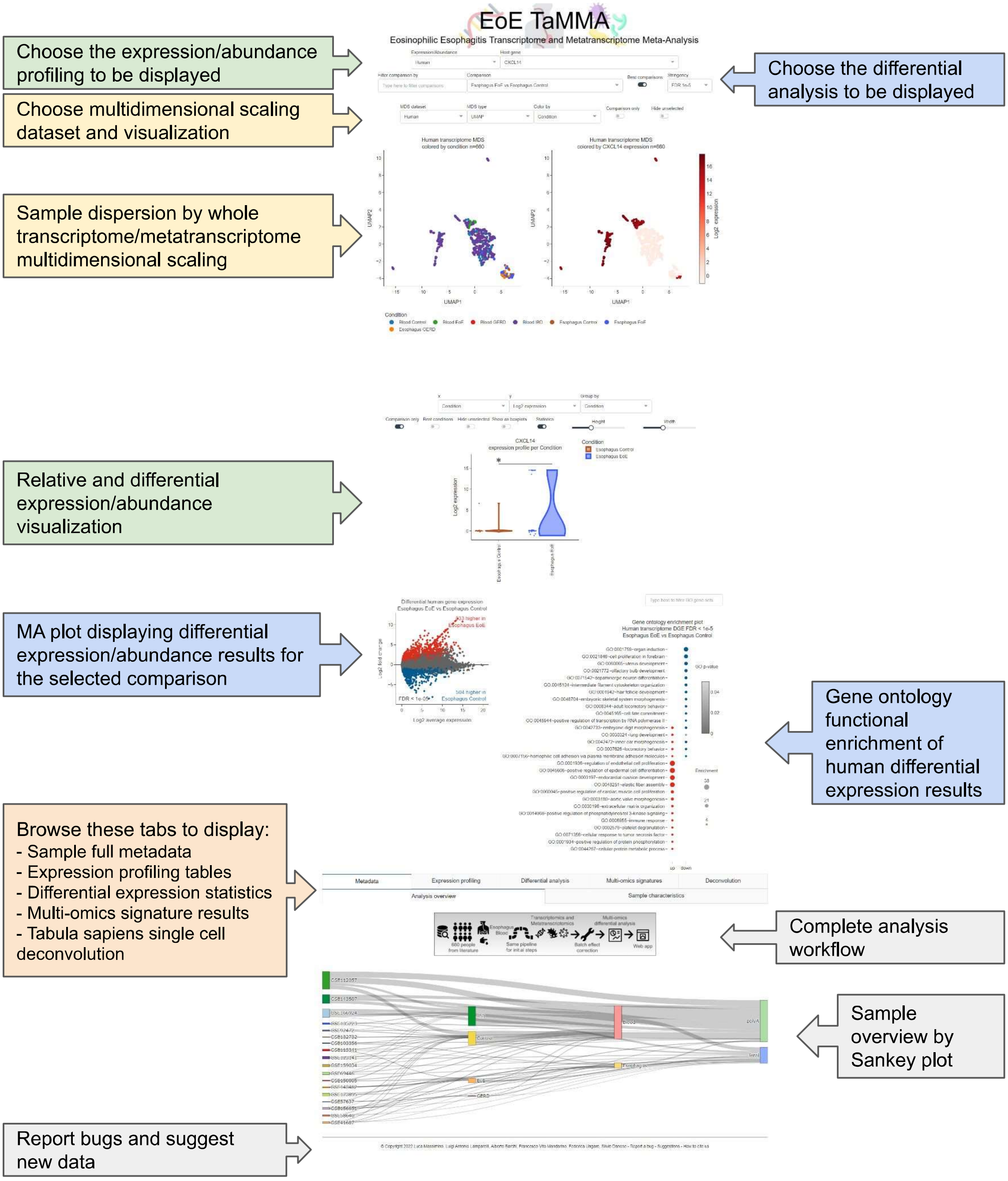

Figure S1. Web app user interface. Global overview of the web app user interface where info boxes are color-coded by category or section, with represents expression profiling in green , multidimensional scaling in yellow , differential analysis and functional enrichment in blue, multi tab panel in orange, and general specification in grey.

Figure S2

A

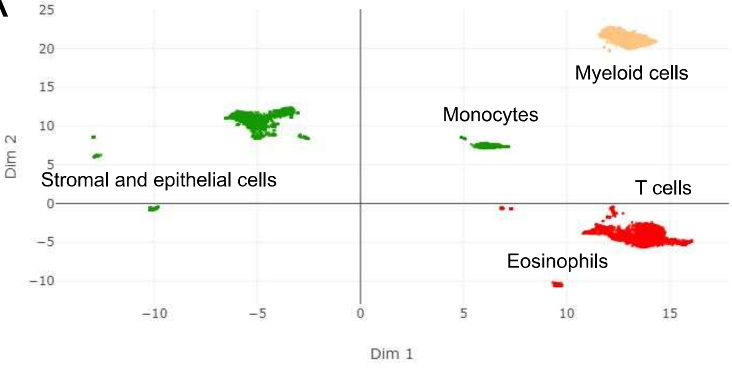

Figure S2. Esophageal mucosa-derived cell populations. (A) UMAP showing color-coded cell populations colonizinf esophageal mucosa according to a single-cell analysis by of Morgan et al., 2021.

Figure S3

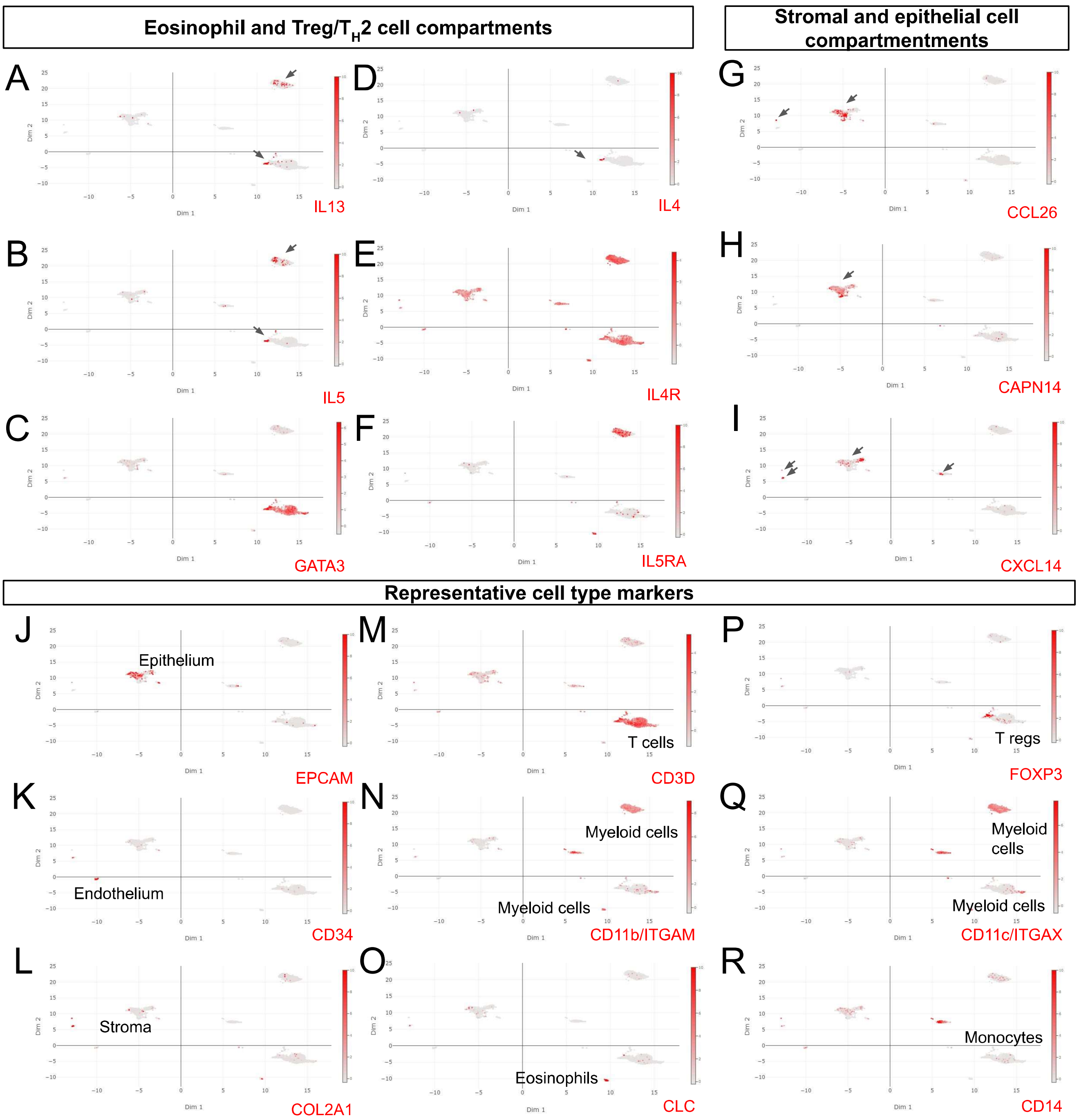

Figure S3. Single-cell analysis of esophageal mucosa. (A-R) UMAP plots showing relative gene expression profiles at a single-cell resolution of Morgan et al., 2021 data. (A-I) Expression profiles of genes found to be differentially expressed in EoE TaMMA. Arrows indicate the clusters with the highest gene expression. (J-N) Expression profiles of representative cell type markers are labeled in black.

Figure S4

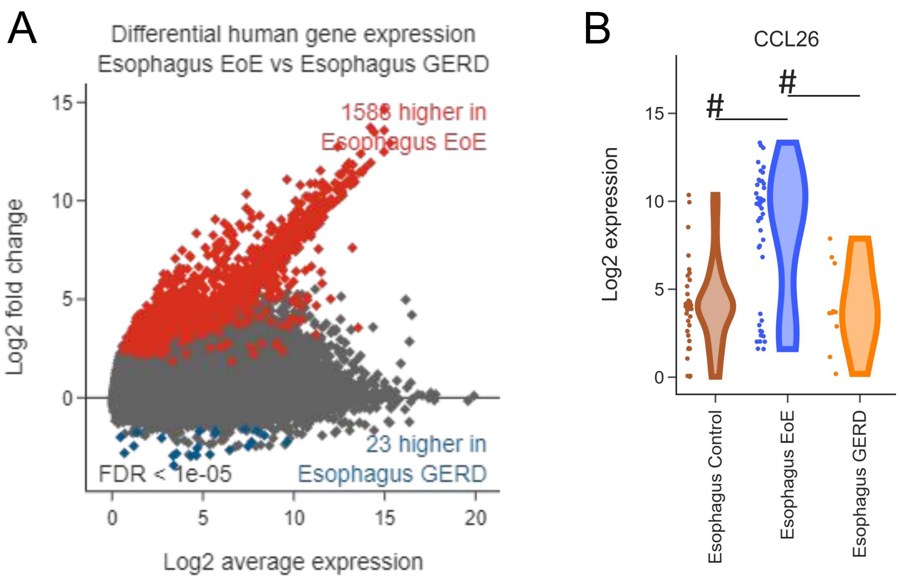

Figure S4. Gene expression profile of EoE and GERD esophagi. (A) MA plot showing the differential gene expression results expressed between the indicated comparisons as a function of log2(average gene expression). Red dots represent genes being differentially expressed with high statistical significance (false discovery rate (FDR) < 1 × 10−5). The number of differentially expressed genes and their trends are red and blue for the up and down-regulated genes, respectively. (**B**) Violin plots showing the differential CCL26 expression among the EoE GERD and control samples. The hashtags indicate FDR < 1 × 10−5.

Figure S5

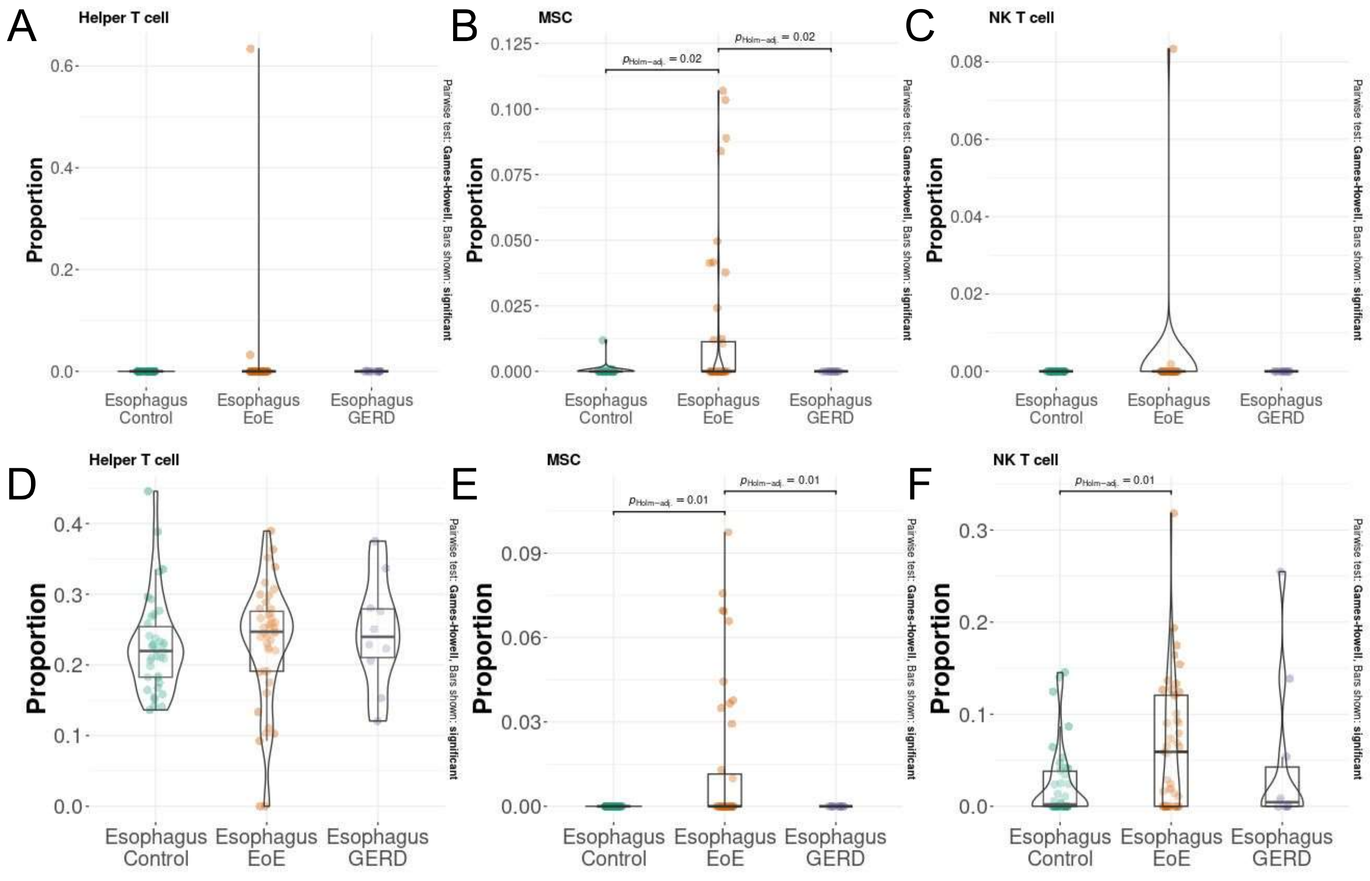

Figure S5. Deconvolution differential analysis. (A-F) Violin/box plots showing the proportions of helper T cells (A,D), mesenchymal stem cells, MSC (B,E), and natural killer, NK cells (C,F) in EoE, GERD, and control esophagi, exploiting MuSiC (A-C) or CIBERSORTx (D-F)..

Figure S6

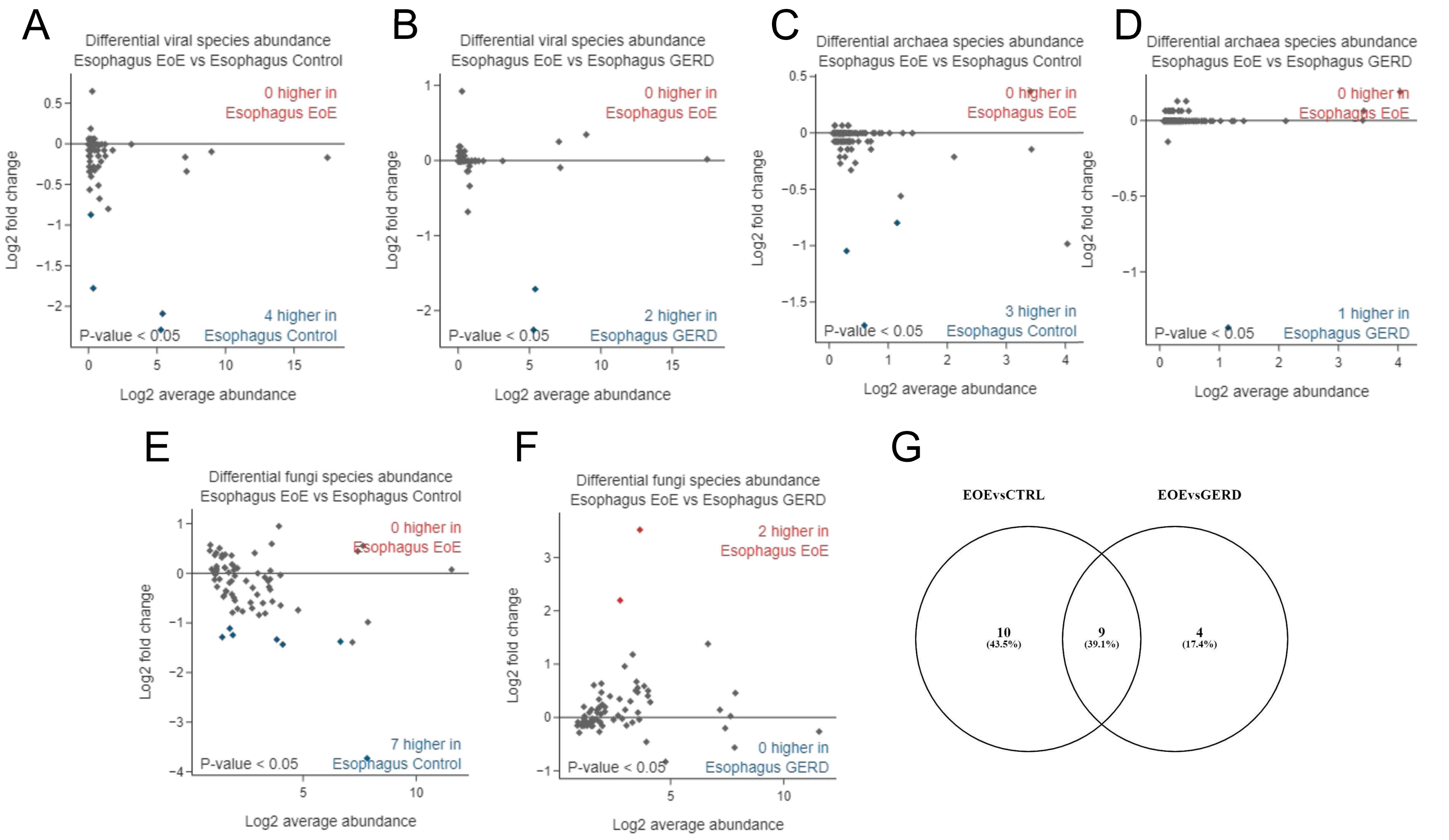

Figure S6. Microbiota profiling of EoE (A-F) MA-plot showing the relative differential abundance of viral (A, B), archaeal (C, D), and fungal (E, F) species between EoE and control and EoE and GERD. Red dots represent microbial species being differentially abundant with high statistical significance (P < 0.05). (**G**) Venn diagram intersecting highly upregulated bacterial species of EoE and GERD.

Figure S7

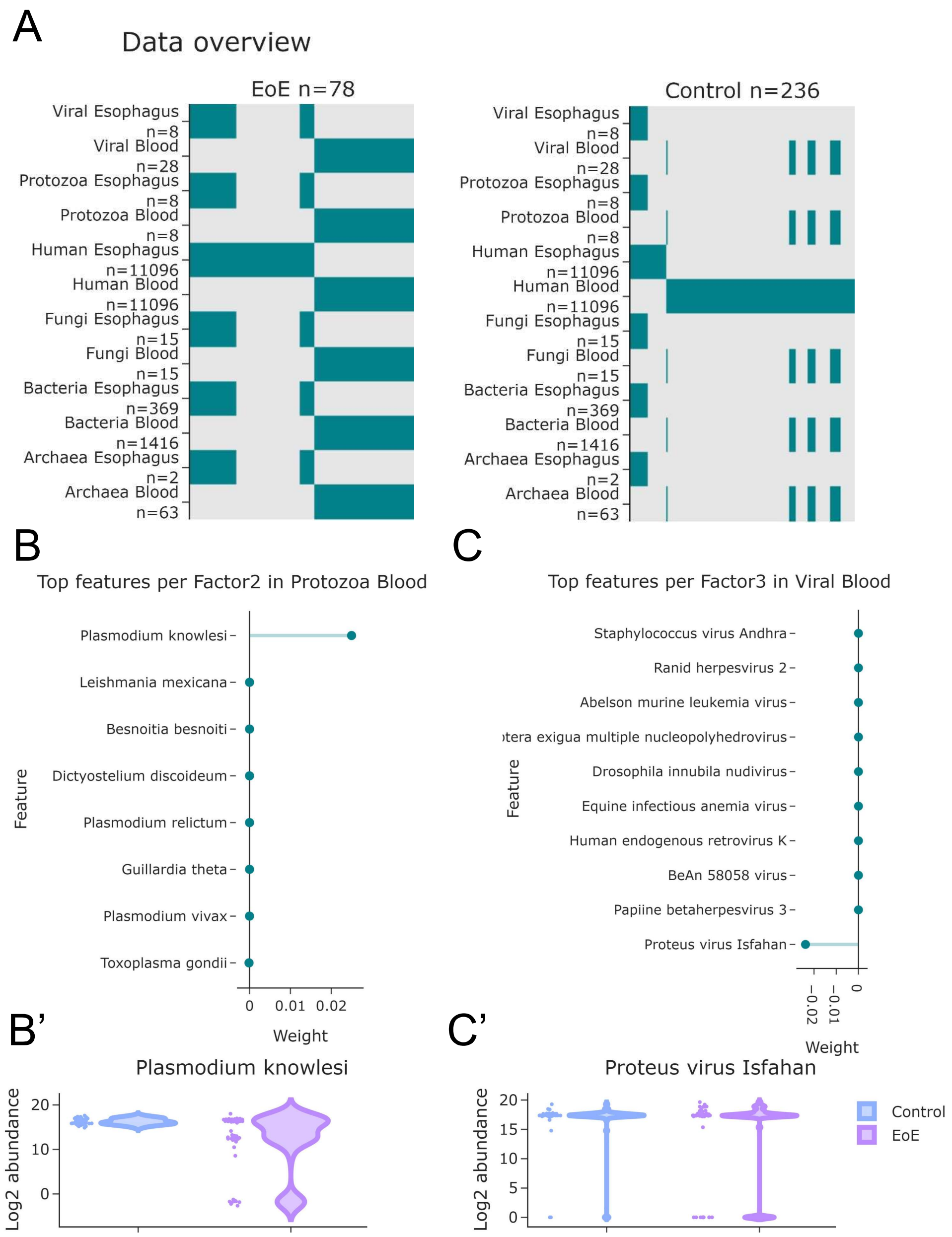

Figure S7. Multi-omic analysis in EoE TaMMA. (**A**) Data overview of MOFA analysis in EoE and control samples, shown as a heatmap where the presence and the absence of data are represented as indigo or light grey cell, respectively. (**B-C**) Needle plots showing weights representing the variance explained by each feature for the indicated factors and layers (B,C) and violin plots showing the relative abundance of top features within conditions (B',C').
