# Supplementary material for: A multi-omic analysis reveals the esophageal dysbiosis as the predominant trait of eosinophilic esophagitis
Source: J Transl Med. 2023 Jan 25;21:46. doi: 10.1186/s12967-023-03898-x (PMC9875471; doi:10.1186/s12967-023-03898-x)
Supplement: Supplementary file 2 — Additional file 2. Additional Table. [file 12967_2023_3898_MOESM2_ESM.pdf]

Sample	Source	Condition	Group	Tissue	Library prep strategy	Library prep kit	Blood rna isolation	Gender	Age	Tissue sublocation	Cell type	Treatment
GSM1022678	GSE41687	Esophagus EoE	EoE	Esophagus	Total	Illumina TruSeq RNA sample prep kit	NA	Male		Proximal	NA	None
GSM1022679	GSE41687	Esophagus EoE	EoE	Esophagus	Total	Illumina TruSeq RNA sample prep kit	NA	Male		Proximal	NA	None
GSM1022680	GSE41687	Esophagus EoE	EoE	Esophagus	Total	Illumina TruSeq RNA sample prep kit	NA	Male		Proximal	NA	None
GSM1022681	GSE41687	Esophagus EoE	EoE	Esophagus	Total	Illumina TruSeq RNA sample prep kit	NA	Male		Distal	NA	None
GSM1022682	GSE41687	Esophagus EoE	EoE	Esophagus	Total	Illumina TruSeq RNA sample prep kit	NA	Male		Distal	NA	None
GSM1022683	GSE41687	Esophagus EoE	EoE	Esophagus	Total	Illumina TruSeq RNA sample prep kit	NA	Male		Distal	NA	None
GSM1022684	GSE41687	Esophagus GERD	GERD	Esophagus	Total	Illumina TruSeq RNA sample prep kit	NA	Male		Proximal	NA	None
GSM1022685	GSE41687	Esophagus GERD	GERD	Esophagus	Total	Illumina TruSeq RNA sample prep kit	NA	Male		Proximal	NA	None
GSM1022686	GSE41687	Esophagus GERD	GERD	Esophagus	Total	Illumina TruSeq RNA sample prep kit	NA	Male		Proximal	NA	None
GSM1022687	GSE41687	Esophagus GERD	GERD	Esophagus	Total	Illumina TruSeq RNA sample prep kit	NA	Male		Distal	NA	None
GSM1022688	GSE41687	Esophagus GERD	GERD	Esophagus	Total	Illumina TruSeq RNA sample prep kit	NA	Male		Distal	NA	None
GSM1022689	GSE41687	Esophagus GERD	GERD	Esophagus	Total	Illumina TruSeq RNA sample prep kit	NA	Male		Distal	NA	None
GSM1022690	GSE41687	Esophagus Control	Control	Esophagus	Total	Illumina TruSeq RNA sample prep kit	NA	Male		Proximal	NA	None
GSM1022691	GSE41687	Esophagus Control	Control	Esophagus	Total	Illumina TruSeq RNA sample prep kit	NA	Male		Proximal	NA	None
GSM1022692	GSE41687	Esophagus Control	Control	Esophagus	Total	Illumina TruSeq RNA sample prep kit	NA	Male		Proximal	NA	None
GSM1022693	GSE41687	Esophagus Control	Control	Esophagus	Total	Illumina TruSeq RNA sample prep kit	NA	Male		Distal	NA	None
GSM1022694	GSE41687	Esophagus Control	Control	Esophagus	Total	Illumina TruSeq RNA sample prep kit	NA	Male		Distal	NA	None
GSM1022695	GSE41687	Esophagus Control	Control	Esophagus	Total	Illumina TruSeq RNA sample prep kit	NA	Male		Distal	NA	None
GSM1385778	GSE57637	Esophagus Control	Control	Esophagus	Total	Illumina TruSeq RNA sample prep kit	NA	NA		NA	ECs TE-7	None
GSM1385779	GSE57637	Esophagus Control	Control	Esophagus	Total	Illumina TruSeq RNA sample prep kit	NA	NA		NA	ECs TE-7	None
GSM1385780	GSE57637	Esophagus Control	Control	Esophagus	Total	Illumina TruSeq RNA sample prep kit	NA	NA		NA	ECs TE-7	None
GSM1385781	GSE57637	Esophagus Control	Control	Esophagus	Total	Illumina TruSeq RNA sample prep kit	NA	NA		NA	ECs TE-7	IL-13
GSM1385782	GSE57637	Esophagus Control	Control	Esophagus	Total	Illumina TruSeq RNA sample prep kit	NA	NA		NA	ECs TE-7	IL-13
GSM1385783	GSE57637	Esophagus Control	Control	Esophagus	Total	Illumina TruSeq RNA sample prep kit	NA	NA		NA	ECs TE-7	IL-13
GSM1415906	GSE58640	Esophagus Control	Control	Esophagus	Total	Illumina TruSeq RNA sample prep kit	NA	NA		NA	NA	None
GSM1415907	GSE58640	Esophagus Control	Control	Esophagus	Total	Illumina TruSeq RNA sample prep kit	NA	NA		NA	NA	None
GSM1415908	GSE58640	Esophagus Control	Control	Esophagus	Total	Illumina TruSeq RNA sample prep kit	NA	NA		NA	NA	None
GSM1415909	GSE58640	Esophagus Control	Control	Esophagus	Total	Illumina TruSeq RNA sample prep kit	NA	NA		NA	NA	None

GSM1415910	GSE58640	Esophagus Control	Control	Esophagus	Total	Illumina TruSeq RNA sample prep kit	NA	NA	NA	NA	None
GSM1415911	GSE58640	Esophagus Control	Control	Esophagus	Total	Illumina TruSeq RNA sample prep kit	NA	NA	NA	NA	None
GSM1415912	GSE58640	Esophagus EoE	EoE	Esophagus	Total	Illumina TruSeq RNA sample prep kit	NA	NA	NA	NA	None
GSM1415913	GSE58640	Esophagus EoE	EoE	Esophagus	Total	Illumina TruSeq RNA sample prep kit	NA	NA	NA	NA	None
GSM1415914	GSE58640	Esophagus EoE	EoE	Esophagus	Total	Illumina TruSeq RNA sample prep kit	NA	NA	NA	NA	None
GSM1415915	GSE58640	Esophagus EoE	EoE	Esophagus	Total	Illumina TruSeq RNA sample prep kit	NA	NA	NA	NA	None
GSM1415916	GSE58640	Esophagus EoE	EoE	Esophagus	Total	Illumina TruSeq RNA sample prep kit	NA	NA	NA	NA	None
GSM1415917	GSE58640	Esophagus EoE	EoE	Esophagus	Total	Illumina TruSeq RNA sample prep kit	NA	NA	NA	NA	None
GSM1415918	GSE58640	Esophagus EoE	EoE	Esophagus	Total	Illumina TruSeq RNA sample prep kit	NA	NA	NA	NA	None
GSM1415919	GSE58640	Esophagus EoE	EoE	Esophagus	Total	Illumina TruSeq RNA sample prep kit	NA	NA	NA	NA	None
GSM1415920	GSE58640	Esophagus EoE	EoE	Esophagus	Total	Illumina TruSeq RNA sample prep kit	NA	NA	NA	NA	None
GSM1415921	GSE58640	Esophagus EoE	EoE	Esophagus	Total	Illumina TruSeq RNA sample prep kit	NA	NA	NA	NA	None
GSM2769390	GSE103356	Esophagus Control	Control	Esophagus	polyA	Illumina TruSeq RNA sample prep kit	NA	NA	NA	ECs EPC2	None
GSM2769391	GSE103356	Esophagus Control	Control	Esophagus	polyA	Illumina TruSeq RNA sample prep kit	NA	NA	NA	ECs EPC2	None
GSM2769392	GSE103356	Esophagus Control	Control	Esophagus	polyA	Illumina TruSeq RNA sample prep kit	NA	NA	NA	ECs EPC2	None
GSM2769393	GSE103356	Esophagus Control	Control	Esophagus	polyA	Illumina TruSeq RNA sample prep kit	NA	NA	NA	ECs EPC2	SPINK7 silencing
GSM2769394	GSE103356	Esophagus Control	Control	Esophagus	polyA	Illumina TruSeq RNA sample prep kit	NA	NA	NA	ECs EPC2	SPINK7 silencing
GSM2769395	GSE103356	Esophagus Control	Control	Esophagus	polyA	Illumina TruSeq RNA sample prep kit	NA	NA	NA	ECs EPC2	SPINK7 silencing
GSM2769396	GSE103356	Esophagus Control	Control	Esophagus	polyA	Illumina TruSeq RNA sample prep kit	NA	NA	NA	ESCs EPC2	None
GSM2769397	GSE103356	Esophagus Control	Control	Esophagus	polyA	Illumina TruSeq RNA sample prep kit	NA	NA	NA	ESCs EPC2	None
GSM2769398	GSE103356	Esophagus Control	Control	Esophagus	polyA	Illumina TruSeq RNA sample prep kit	NA	NA	NA	ESCs EPC2	None
GSM3103946	GSE113341	Esophagus EoE	EoE	Esophagus	polyA	Illumina TruSeq RNA sample prep kit	NA	Male	Distal	NA	None
GSM3103947	GSE113341	Esophagus EoE	EoE	Esophagus	polyA	Illumina TruSeq RNA sample prep kit	NA	Male	Distal	NA	None
GSM3103948	GSE113341	Esophagus EoE	EoE	Esophagus	polyA	Illumina TruSeq RNA sample prep kit	NA	Male	Distal	NA	None
GSM3103949	GSE113341	Esophagus EoE	EoE	Esophagus	polyA	Illumina TruSeq RNA sample prep kit	NA	Male	Distal	NA	None
GSM3103950	GSE113341	Esophagus EoE	EoE	Esophagus	polyA	Illumina TruSeq RNA sample prep kit	NA	Male	Distal	NA	None
GSM3103951	GSE113341	Esophagus EoE	EoE	Esophagus	polyA	Illumina TruSeq RNA sample prep kit	NA	Female	Distal	NA	None
GSM3103952	GSE113341	Esophagus EoE	EoE	Esophagus	polyA	Illumina TruSeq RNA sample prep kit	NA	Female	Distal	NA	None
GSM3103953	GSE113341	Esophagus EoE	EoE	Esophagus	polyA	Illumina TruSeq RNA sample prep kit	NA	Female	Distal	NA	None

GSM3103954	GSE113341	Esophagus EoE	EoE	Esophagus	polyA	Illumina TruSeq RNA sample prep kit	NA	Female		Distal	NA	None
GSM3103955	GSE113341	Esophagus EoE	EoE	Esophagus	polyA	Illumina TruSeq RNA sample prep kit	NA	Female		Distal	NA	None
GSM3103956	GSE113341	Esophagus Control	Control	Esophagus	polyA	Illumina TruSeq RNA sample prep kit	NA	Male		Distal	NA	None
GSM3103957	GSE113341	Esophagus Control	Control	Esophagus	polyA	Illumina TruSeq RNA sample prep kit	NA	Male		Distal	NA	None
GSM3103958	GSE113341	Esophagus Control	Control	Esophagus	polyA	Illumina TruSeq RNA sample prep kit	NA	Male		Distal	NA	None
GSM3103959	GSE113341	Esophagus Control	Control	Esophagus	polyA	Illumina TruSeq RNA sample prep kit	NA	Male		Distal	NA	None
GSM3103960	GSE113341	Esophagus Control	Control	Esophagus	polyA	Illumina TruSeq RNA sample prep kit	NA	Male		Distal	NA	None
GSM3103961	GSE113341	Esophagus Control	Control	Esophagus	polyA	Illumina TruSeq RNA sample prep kit	NA	Female		Distal	NA	None
GSM3103962	GSE113341	Esophagus Control	Control	Esophagus	polyA	Illumina TruSeq RNA sample prep kit	NA	Female		Distal	NA	None
GSM3103963	GSE113341	Esophagus Control	Control	Esophagus	polyA	Illumina TruSeq RNA sample prep kit	NA	Female		Distal	NA	None
GSM3103964	GSE113341	Esophagus Control	Control	Esophagus	polyA	Illumina TruSeq RNA sample prep kit	NA	Female		Distal	NA	None
GSM3103965	GSE113341	Esophagus Control	Control	Esophagus	polyA	Illumina TruSeq RNA sample prep kit	NA	Female		Distal	NA	None
GSM4260265	GSE143482	Esophagus EoE	EoE	Esophagus	polyA	NEBNext Ultra II Directional RNA Library Prep Kit	NA	NA		NA	Fibroblasts	TNFSF14
GSM4260266	GSE143482	Esophagus EoE	EoE	Esophagus	polyA	NEBNext Ultra II Directional RNA Library Prep Kit	NA	NA		NA	Fibroblasts	TGFB1
GSM4260267	GSE143482	Esophagus EoE	EoE	Esophagus	polyA	NEBNext Ultra II Directional RNA Library Prep Kit	NA	NA		NA	Fibroblasts	None
GSM4260268	GSE143482	Esophagus EoE	EoE	Esophagus	polyA	NEBNext Ultra II Directional RNA Library Prep Kit	NA	NA		NA	Fibroblasts	TNFSF14
GSM4260269	GSE143482	Esophagus EoE	EoE	Esophagus	polyA	NEBNext Ultra II Directional RNA Library Prep Kit	NA	NA		NA	Fibroblasts	TGFB1
GSM4260270	GSE143482	Esophagus EoE	EoE	Esophagus	polyA	NEBNext Ultra II Directional RNA Library Prep Kit	NA	NA		NA	Fibroblasts	None
GSM4260271	GSE143482	Esophagus EoE	EoE	Esophagus	polyA	NEBNext Ultra II Directional RNA Library Prep Kit	NA	NA		NA	Fibroblasts	TNFSF14
GSM4260272	GSE143482	Esophagus EoE	EoE	Esophagus	polyA	NEBNext Ultra II Directional RNA Library Prep Kit	NA	NA		NA	Fibroblasts	TGFB1
GSM4260273	GSE143482	Esophagus EoE	EoE	Esophagus	polyA	NEBNext Ultra II Directional RNA Library Prep Kit	NA	NA		NA	Fibroblasts	None
GSM4260274	GSE143482	Esophagus EoE	EoE	Esophagus	polyA	NEBNext Ultra II Directional RNA Library Prep Kit	NA	NA		NA	Fibroblasts	TNFSF14
GSM4260275	GSE143482	Esophagus EoE	EoE	Esophagus	polyA	NEBNext Ultra II Directional RNA Library Prep Kit	NA	NA		NA	Fibroblasts	TGFB1

GSM4260276	GSE143482	Esophagus EoE	EoE	Esophagus	polyA	NEBNext Ultra II Directional RNA Library Prep Kit	NA	NA		NA	Fibroblasts	None
GSM4735785	GSE156651	Blood EoE	EoE	Blood	Total	SMART-Seq v4 Ultra Low Input RNA Kit	Tempus Blood RNA Tubes	NA	75	NA	NA	None
GSM4735786	GSE156651	Blood Control	Control	Blood	Total	SMART-Seq v4 Ultra Low Input RNA Kit	Tempus Blood RNA Tubes	NA	67	NA	NA	None
GSM4735787	GSE156651	Blood EoE	EoE	Blood	Total	SMART-Seq v4 Ultra Low Input RNA Kit	Tempus Blood RNA Tubes	NA	34	NA	NA	None
GSM4735788	GSE156651	Blood GERD	GERD	Blood	Total	SMART-Seq v4 Ultra Low Input RNA Kit	Tempus Blood RNA Tubes	NA	39	NA	NA	None
GSM4735789	GSE156651	Blood GERD	GERD	Blood	Total	SMART-Seq v4 Ultra Low Input RNA Kit	Tempus Blood RNA Tubes	NA	38	NA	NA	None
GSM4735790	GSE156651	Blood GERD	GERD	Blood	Total	SMART-Seq v4 Ultra Low Input RNA Kit	Tempus Blood RNA Tubes	NA	60	NA	NA	None
GSM4735791	GSE156651	Blood GERD	GERD	Blood	Total	SMART-Seq v4 Ultra Low Input RNA Kit	Tempus Blood RNA Tubes	NA	65	NA	NA	None
GSM4735792	GSE156651	Blood EoE	EoE	Blood	Total	SMART-Seq v4 Ultra Low Input RNA Kit	Tempus Blood RNA Tubes	NA	32	NA	NA	None
GSM4735793	GSE156651	Blood EoE	EoE	Blood	Total	SMART-Seq v4 Ultra Low Input RNA Kit	Tempus Blood RNA Tubes	NA	51	NA	NA	None
GSM4735794	GSE156651	Blood EoE	EoE	Blood	Total	SMART-Seq v4 Ultra Low Input RNA Kit	Tempus Blood RNA Tubes	NA	25	NA	NA	None
GSM4735795	GSE156651	Esophagus EoE	EoE	Esophagus	Total	SMART-Seq v4 Ultra Low Input RNA Kit	NA	NA	75	NA	NA	None
GSM4735796	GSE156651	Esophagus Control	Control	Esophagus	Total	SMART-Seq v4 Ultra Low Input RNA Kit	NA	NA	67	NA	NA	None
GSM4735797	GSE156651	Esophagus EoE	EoE	Esophagus	Total	SMART-Seq v4 Ultra Low Input RNA Kit	NA	NA	34	NA	NA	None
GSM4735798	GSE156651	Esophagus GERD	GERD	Esophagus	Total	SMART-Seq v4 Ultra Low Input RNA Kit	NA	NA	39	NA	NA	None
GSM4735799	GSE156651	Esophagus GERD	GERD	Esophagus	Total	SMART-Seq v4 Ultra Low Input RNA Kit	NA	NA	38	NA	NA	None
GSM4735800	GSE156651	Esophagus GERD	GERD	Esophagus	Total	SMART-Seq v4 Ultra Low Input RNA Kit	NA	NA	60	NA	NA	None
GSM4735801	GSE156651	Esophagus GERD	GERD	Esophagus	Total	SMART-Seq v4 Ultra Low Input RNA Kit	NA	NA	65	NA	NA	None
GSM4735802	GSE156651	Esophagus EoE	EoE	Esophagus	Total	SMART-Seq v4 Ultra Low Input RNA Kit	NA	NA	32	NA	NA	None

GSM4735803	GSE156651	Esophagus EoE	EoE	Esophagus	Total	SMART-Seq v4 Ultra Low Input RNA Kit	NA	NA	51	NA	NA	None
GSM4735804	GSE156651	Esophagus EoE	EoE	Esophagus	Total	SMART-Seq v4 Ultra Low Input RNA Kit	NA	NA	25	NA	NA	None
GSM5282507	GSE173895	Blood EoE	EoE	Blood	Total	Ovation Ultralow Library System V2	Invitrogen Dynabeads	NA	11	NA	CD4+	None
GSM5282508	GSE173895	Blood EoE	EoE	Blood	Total	Ovation Ultralow Library System V2	Invitrogen Dynabeads	NA	11	NA	CD4+	None
GSM5282509	GSE173895	Blood EoE	EoE	Blood	Total	Ovation Ultralow Library System V2	Invitrogen Dynabeads	NA	11	NA	CD4+	EPIT
GSM5282510	GSE173895	Blood EoE	EoE	Blood	Total	Ovation Ultralow Library System V2	Invitrogen Dynabeads	NA	9	NA	CD4+	None
GSM5282511	GSE173895	Blood EoE	EoE	Blood	Total	Ovation Ultralow Library System V2	Invitrogen Dynabeads	NA	9	NA	CD4+	None
GSM5282512	GSE173895	Blood EoE	EoE	Blood	Total	Ovation Ultralow Library System V2	Invitrogen Dynabeads	NA	13	NA	CD4+	None
GSM5282513	GSE173895	Blood EoE	EoE	Blood	Total	Ovation Ultralow Library System V2	Invitrogen Dynabeads	NA	13	NA	CD4+	EPIT
GSM5282514	GSE173895	Blood EoE	EoE	Blood	Total	Ovation Ultralow Library System V2	Invitrogen Dynabeads	NA	12	NA	CD4+	None
GSM5282515	GSE173895	Blood EoE	EoE	Blood	Total	Ovation Ultralow Library System V2	Invitrogen Dynabeads	NA	12	NA	CD4+	Placebo
GSM5282516	GSE173895	Blood EoE	EoE	Blood	Total	Ovation Ultralow Library System V2	Invitrogen Dynabeads	NA	8	NA	CD4+	None
GSM5282517	GSE173895	Blood EoE	EoE	Blood	Total	Ovation Ultralow Library System V2	Invitrogen Dynabeads	NA	8	NA	CD4+	None
GSM5282518	GSE173895	Blood EoE	EoE	Blood	Total	Ovation Ultralow Library System V2	Invitrogen Dynabeads	NA	8	NA	CD4+	EPIT
GSM5282519	GSE173895	Blood EoE	EoE	Blood	Total	Ovation Ultralow Library System V2	Invitrogen Dynabeads	NA	12	NA	CD4+	None
GSM5282520	GSE173895	Blood EoE	EoE	Blood	Total	Ovation Ultralow Library System V2	Invitrogen Dynabeads	NA	12	NA	CD4+	None
GSM5282521	GSE173895	Blood EoE	EoE	Blood	Total	Ovation Ultralow Library System V2	Invitrogen Dynabeads	NA	5	NA	CD4+	None
GSM5282522	GSE173895	Blood EoE	EoE	Blood	Total	Ovation Ultralow Library System V2	Invitrogen Dynabeads	NA	5	NA	CD4+	None
GSM5282523	GSE173895	Blood EoE	EoE	Blood	Total	Ovation Ultralow Library System V2	Invitrogen Dynabeads	NA	5	NA	CD4+	EPIT
GSM5282524	GSE173895	Blood EoE	EoE	Blood	Total	Ovation Ultralow Library System V2	Invitrogen Dynabeads	NA	8	NA	CD4+	EPIT
GSM5282525	GSE173895	Blood EoE	EoE	Blood	Total	Ovation Ultralow Library System V2	Invitrogen Dynabeads	NA	15	NA	CD4+	EPIT
GSM5282526	GSE173895	Blood EoE	EoE	Blood	Total	Ovation Ultralow Library System V2	Invitrogen Dynabeads	NA	6	NA	CD4+	None
GSM5282527	GSE173895	Blood EoE	EoE	Blood	Total	Ovation Ultralow Library System V2	Invitrogen Dynabeads	NA	6	NA	CD4+	EPIT
GSM5282528	GSE173895	Blood EoE	EoE	Blood	Total	Ovation Ultralow Library System V2	Invitrogen Dynabeads	NA	12	NA	CD4+	None
GSM5282529	GSE173895	Blood EoE	EoE	Blood	Total	Ovation Ultralow Library System V2	Invitrogen Dynabeads	NA	12	NA	CD4+	Placebo
GSM5282530	GSE173895	Blood EoE	EoE	Blood	Total	Ovation Ultralow Library System V2	Invitrogen Dynabeads	NA	15	NA	CD4+	None
GSM5282531	GSE173895	Blood EoE	EoE	Blood	Total	Ovation Ultralow Library System V2	Invitrogen Dynabeads	NA	15	NA	CD4+	None
GSM5282532	GSE173895	Blood EoE	EoE	Blood	Total	Ovation Ultralow Library System V2	Invitrogen Dynabeads	NA	8	NA	CD4+	None

GSM5282533	GSE173895	Blood EoE	EoE	Blood	Total	Ovation Ultralow Library System V2	Invitrogen Dynabeads	NA	8	NA	CD4+	EPIT
GSM5282534	GSE173895	Blood EoE	EoE	Blood	Total	Ovation Ultralow Library System V2	Invitrogen Dynabeads	NA	7	NA	CD4+	EPIT
GSM5282535	GSE173895	Blood EoE	EoE	Blood	Total	Ovation Ultralow Library System V2	Invitrogen Dynabeads	NA	14	NA	CD4+	Placebo
GSM5282536	GSE173895	Blood EoE	EoE	Blood	Total	Ovation Ultralow Library System V2	Invitrogen Dynabeads	NA	11	NA	CD4+	EPIT
GSM4260927	GSE143507	Blood IBD	IBD	Blood	polyA	Illumina TruSeq RNA sample prep kit	PAXgene Blood RNA kit	Male	28	NA	NA	None
GSM4260928	GSE143507	Blood IBD	IBD	Blood	polyA	Illumina TruSeq RNA sample prep kit	PAXgene Blood RNA kit	Female	35	NA	NA	None
GSM4260929	GSE143507	Blood IBD	IBD	Blood	polyA	Illumina TruSeq RNA sample prep kit	PAXgene Blood RNA kit	Female	39	NA	NA	None
GSM4260930	GSE143507	Blood IBD	IBD	Blood	polyA	Illumina TruSeq RNA sample prep kit	PAXgene Blood RNA kit	Male	32	NA	NA	None
GSM4260931	GSE143507	Blood IBD	IBD	Blood	polyA	Illumina TruSeq RNA sample prep kit	PAXgene Blood RNA kit	Male	47	NA	NA	None
GSM4260932	GSE143507	Blood IBD	IBD	Blood	polyA	Illumina TruSeq RNA sample prep kit	PAXgene Blood RNA kit	Female	41	NA	NA	None
GSM4260933	GSE143507	Blood IBD	IBD	Blood	polyA	Illumina TruSeq RNA sample prep kit	PAXgene Blood RNA kit	Male	45	NA	NA	None
GSM4260934	GSE143507	Blood IBD	IBD	Blood	polyA	Illumina TruSeq RNA sample prep kit	PAXgene Blood RNA kit	Male	49	NA	NA	None
GSM4260935	GSE143507	Blood IBD	IBD	Blood	polyA	Illumina TruSeq RNA sample prep kit	PAXgene Blood RNA kit	Male	35	NA	NA	None
GSM4260936	GSE143507	Blood IBD	IBD	Blood	polyA	Illumina TruSeq RNA sample prep kit	PAXgene Blood RNA kit	Female	53	NA	NA	None
GSM4260937	GSE143507	Blood IBD	IBD	Blood	polyA	Illumina TruSeq RNA sample prep kit	PAXgene Blood RNA kit	Male	48	NA	NA	None
GSM4260938	GSE143507	Blood IBD	IBD	Blood	polyA	Illumina TruSeq RNA sample prep kit	PAXgene Blood RNA kit	Female	43	NA	NA	None
GSM4260939	GSE143507	Blood IBD	IBD	Blood	polyA	Illumina TruSeq RNA sample prep kit	PAXgene Blood RNA kit	Male	36	NA	NA	None
GSM4260940	GSE143507	Blood IBD	IBD	Blood	polyA	Illumina TruSeq RNA sample prep kit	PAXgene Blood RNA kit	Female	37	NA	NA	None
GSM4260941	GSE143507	Blood IBD	IBD	Blood	polyA	Illumina TruSeq RNA sample prep kit	PAXgene Blood RNA kit	Male	41	NA	NA	None
GSM4260942	GSE143507	Blood IBD	IBD	Blood	polyA	Illumina TruSeq RNA sample prep kit	PAXgene Blood RNA kit	Male	54	NA	NA	None
GSM4260943	GSE143507	Blood IBD	IBD	Blood	polyA	Illumina TruSeq RNA sample prep kit	PAXgene Blood RNA kit	Male	49	NA	NA	None
GSM4260944	GSE143507	Blood IBD	IBD	Blood	polyA	Illumina TruSeq RNA sample prep kit	PAXgene Blood RNA kit	Male	58	NA	NA	None
GSM4260945	GSE143507	Blood IBD	IBD	Blood	polyA	Illumina TruSeq RNA sample prep kit	PAXgene Blood RNA kit	Female	34	NA	NA	None
GSM4260946	GSE143507	Blood IBD	IBD	Blood	polyA	Illumina TruSeq RNA sample prep kit	PAXgene Blood RNA kit	Female	46	NA	NA	None
GSM4260947	GSE143507	Blood IBD	IBD	Blood	polyA	Illumina TruSeq RNA sample prep kit	PAXgene Blood RNA kit	Male	41	NA	NA	None
GSM4260948	GSE143507	Blood IBD	IBD	Blood	polyA	Illumina TruSeq RNA sample prep kit	PAXgene Blood RNA kit	Male	54	NA	NA	None
GSM4260949	GSE143507	Blood IBD	IBD	Blood	polyA	Illumina TruSeq RNA sample prep kit	PAXgene Blood RNA kit	Male	35	NA	NA	None
GSM4260950	GSE143507	Blood IBD	IBD	Blood	polyA	Illumina TruSeq RNA sample prep kit	PAXgene Blood RNA kit	Male	34	NA	NA	None
GSM4260951	GSE143507	Blood IBD	IBD	Blood	polyA	Illumina TruSeq RNA sample prep kit	PAXgene Blood RNA kit	Male	44	NA	NA	None

GSM4260952	GSE143507	Blood IBD	IBD	Blood	polyA	Illumina TruSeq RNA sample prep kit	PAXgene Blood RNA kit	Male	31	NA	NA	None
GSM4260953	GSE143507	Blood IBD	IBD	Blood	polyA	Illumina TruSeq RNA sample prep kit	PAXgene Blood RNA kit	Male	60	NA	NA	None
GSM4260954	GSE143507	Blood IBD	IBD	Blood	polyA	Illumina TruSeq RNA sample prep kit	PAXgene Blood RNA kit	Female	33	NA	NA	None
GSM4260955	GSE143507	Blood IBD	IBD	Blood	polyA	Illumina TruSeq RNA sample prep kit	PAXgene Blood RNA kit	Female	36	NA	NA	None
GSM4260956	GSE143507	Blood IBD	IBD	Blood	polyA	Illumina TruSeq RNA sample prep kit	PAXgene Blood RNA kit	Male	37	NA	NA	None
GSM4260957	GSE143507	Blood IBD	IBD	Blood	polyA	Illumina TruSeq RNA sample prep kit	PAXgene Blood RNA kit	Male	37	NA	NA	None
GSM4260958	GSE143507	Blood IBD	IBD	Blood	polyA	Illumina TruSeq RNA sample prep kit	PAXgene Blood RNA kit	Female	58	NA	NA	None
GSM4260959	GSE143507	Blood IBD	IBD	Blood	polyA	Illumina TruSeq RNA sample prep kit	PAXgene Blood RNA kit	Male	47	NA	NA	None
GSM4260960	GSE143507	Blood IBD	IBD	Blood	polyA	Illumina TruSeq RNA sample prep kit	PAXgene Blood RNA kit	Male	59	NA	NA	None
GSM4260961	GSE143507	Blood IBD	IBD	Blood	polyA	Illumina TruSeq RNA sample prep kit	PAXgene Blood RNA kit	Male	49	NA	NA	None
GSM4260962	GSE143507	Blood IBD	IBD	Blood	polyA	Illumina TruSeq RNA sample prep kit	PAXgene Blood RNA kit	Female	42	NA	NA	None
GSM4260963	GSE143507	Blood IBD	IBD	Blood	polyA	Illumina TruSeq RNA sample prep kit	PAXgene Blood RNA kit	Female	37	NA	NA	None
GSM4260964	GSE143507	Blood IBD	IBD	Blood	polyA	Illumina TruSeq RNA sample prep kit	PAXgene Blood RNA kit	Male	43	NA	NA	None
GSM4260965	GSE143507	Blood IBD	IBD	Blood	polyA	Illumina TruSeq RNA sample prep kit	PAXgene Blood RNA kit	Male	36	NA	NA	None
GSM4260966	GSE143507	Blood IBD	IBD	Blood	polyA	Illumina TruSeq RNA sample prep kit	PAXgene Blood RNA kit	Male	36	NA	NA	None
GSM4260967	GSE143507	Blood IBD	IBD	Blood	polyA	Illumina TruSeq RNA sample prep kit	PAXgene Blood RNA kit	Female	28	NA	NA	None
GSM4260968	GSE143507	Blood IBD	IBD	Blood	polyA	Illumina TruSeq RNA sample prep kit	PAXgene Blood RNA kit	Female	41	NA	NA	None
GSM4260969	GSE143507	Blood IBD	IBD	Blood	polyA	Illumina TruSeq RNA sample prep kit	PAXgene Blood RNA kit	Female	41	NA	NA	None
GSM4260970	GSE143507	Blood IBD	IBD	Blood	polyA	Illumina TruSeq RNA sample prep kit	PAXgene Blood RNA kit	Male	48	NA	NA	None
GSM4260971	GSE143507	Blood IBD	IBD	Blood	polyA	Illumina TruSeq RNA sample prep kit	PAXgene Blood RNA kit	Male	36	NA	NA	None
GSM4260972	GSE143507	Blood IBD	IBD	Blood	polyA	Illumina TruSeq RNA sample prep kit	PAXgene Blood RNA kit	Female	54	NA	NA	None
GSM4260973	GSE143507	Blood IBD	IBD	Blood	polyA	Illumina TruSeq RNA sample prep kit	PAXgene Blood RNA kit	Female	35	NA	NA	None
GSM4260974	GSE143507	Blood IBD	IBD	Blood	polyA	Illumina TruSeq RNA sample prep kit	PAXgene Blood RNA kit	Female	50	NA	NA	None
GSM4260975	GSE143507	Blood IBD	IBD	Blood	polyA	Illumina TruSeq RNA sample prep kit	PAXgene Blood RNA kit	Male	36	NA	NA	None
GSM4260976	GSE143507	Blood IBD	IBD	Blood	polyA	Illumina TruSeq RNA sample prep kit	PAXgene Blood RNA kit	Female	42	NA	NA	None
GSM4260977	GSE143507	Blood IBD	IBD	Blood	polyA	Illumina TruSeq RNA sample prep kit	PAXgene Blood RNA kit	Male	45	NA	NA	None
GSM4260978	GSE143507	Blood IBD	IBD	Blood	polyA	Illumina TruSeq RNA sample prep kit	PAXgene Blood RNA kit	Male	32	NA	NA	None
GSM4260979	GSE143507	Blood IBD	IBD	Blood	polyA	Illumina TruSeq RNA sample prep kit	PAXgene Blood RNA kit	Male	47	NA	NA	None
GSM4260980	GSE143507	Blood IBD	IBD	Blood	polyA	Illumina TruSeq RNA sample prep kit	PAXgene Blood RNA kit	Male	27	NA	NA	None

GSM4260981	GSE143507	Blood IBD	IBD	Blood	polyA	Illumina TruSeq RNA sample prep kit	PAXgene Blood RNA kit	Male	35	NA	NA	None
GSM4260982	GSE143507	Blood IBD	IBD	Blood	polyA	Illumina TruSeq RNA sample prep kit	PAXgene Blood RNA kit	Male	44	NA	NA	None
GSM4260983	GSE143507	Blood IBD	IBD	Blood	polyA	Illumina TruSeq RNA sample prep kit	PAXgene Blood RNA kit	Female	41	NA	NA	None
GSM4260984	GSE143507	Blood IBD	IBD	Blood	polyA	Illumina TruSeq RNA sample prep kit	PAXgene Blood RNA kit	Male	36	NA	NA	None
GSM4260985	GSE143507	Blood IBD	IBD	Blood	polyA	Illumina TruSeq RNA sample prep kit	PAXgene Blood RNA kit	Female	44	NA	NA	None
GSM4260986	GSE143507	Blood IBD	IBD	Blood	polyA	Illumina TruSeq RNA sample prep kit	PAXgene Blood RNA kit	Female	38	NA	NA	None
GSM4260987	GSE143507	Blood IBD	IBD	Blood	polyA	Illumina TruSeq RNA sample prep kit	PAXgene Blood RNA kit	Male	41	NA	NA	None
GSM4260988	GSE143507	Blood IBD	IBD	Blood	polyA	Illumina TruSeq RNA sample prep kit	PAXgene Blood RNA kit	Female	39	NA	NA	None
GSM4260989	GSE143507	Blood IBD	IBD	Blood	polyA	Illumina TruSeq RNA sample prep kit	PAXgene Blood RNA kit	Male	39	NA	NA	None
GSM4260990	GSE143507	Blood IBD	IBD	Blood	polyA	Illumina TruSeq RNA sample prep kit	PAXgene Blood RNA kit	Female	33	NA	NA	None
GSM4260991	GSE143507	Blood IBD	IBD	Blood	polyA	Illumina TruSeq RNA sample prep kit	PAXgene Blood RNA kit	Male	34	NA	NA	None
GSM4260992	GSE143507	Blood IBD	IBD	Blood	polyA	Illumina TruSeq RNA sample prep kit	PAXgene Blood RNA kit	Female	40	NA	NA	None
GSM4260993	GSE143507	Blood IBD	IBD	Blood	polyA	Illumina TruSeq RNA sample prep kit	PAXgene Blood RNA kit	Female	42	NA	NA	None
GSM4260994	GSE143507	Blood IBD	IBD	Blood	polyA	Illumina TruSeq RNA sample prep kit	PAXgene Blood RNA kit	Male	35	NA	NA	None
GSM4260995	GSE143507	Blood IBD	IBD	Blood	polyA	Illumina TruSeq RNA sample prep kit	PAXgene Blood RNA kit	Male	35	NA	NA	None
GSM4260996	GSE143507	Blood IBD	IBD	Blood	polyA	Illumina TruSeq RNA sample prep kit	PAXgene Blood RNA kit	Female	29	NA	NA	None
GSM4260997	GSE143507	Blood IBD	IBD	Blood	polyA	Illumina TruSeq RNA sample prep kit	PAXgene Blood RNA kit	Male	34	NA	NA	None
GSM4260998	GSE143507	Blood IBD	IBD	Blood	polyA	Illumina TruSeq RNA sample prep kit	PAXgene Blood RNA kit	Female	46	NA	NA	None
GSM4260999	GSE143507	Blood IBD	IBD	Blood	polyA	Illumina TruSeq RNA sample prep kit	PAXgene Blood RNA kit	Male	28	NA	NA	None
GSM4261000	GSE143507	Blood IBD	IBD	Blood	polyA	Illumina TruSeq RNA sample prep kit	PAXgene Blood RNA kit	Female	50	NA	NA	None
GSM4261001	GSE143507	Blood IBD	IBD	Blood	polyA	Illumina TruSeq RNA sample prep kit	PAXgene Blood RNA kit	Male	42	NA	NA	None
GSM4261002	GSE143507	Blood IBD	IBD	Blood	polyA	Illumina TruSeq RNA sample prep kit	PAXgene Blood RNA kit	Male	32	NA	NA	None
GSM4261003	GSE143507	Blood IBD	IBD	Blood	polyA	Illumina TruSeq RNA sample prep kit	PAXgene Blood RNA kit	Female	35	NA	NA	None
GSM4261004	GSE143507	Blood IBD	IBD	Blood	polyA	Illumina TruSeq RNA sample prep kit	PAXgene Blood RNA kit	Male	35	NA	NA	None
GSM4261005	GSE143507	Blood IBD	IBD	Blood	polyA	Illumina TruSeq RNA sample prep kit	PAXgene Blood RNA kit	Male	34	NA	NA	None
GSM4261006	GSE143507	Blood IBD	IBD	Blood	polyA	Illumina TruSeq RNA sample prep kit	PAXgene Blood RNA kit	Male	40	NA	NA	None
GSM4261007	GSE143507	Blood IBD	IBD	Blood	polyA	Illumina TruSeq RNA sample prep kit	PAXgene Blood RNA kit	Male	30	NA	NA	None
GSM4261008	GSE143507	Blood IBD	IBD	Blood	polyA	Illumina TruSeq RNA sample prep kit	PAXgene Blood RNA kit	Female	38	NA	NA	None
GSM4261009	GSE143507	Blood IBD	IBD	Blood	polyA	Illumina TruSeq RNA sample prep kit	PAXgene Blood RNA kit	Female	39	NA	NA	None

GSM4261010	GSE143507	Blood IBD	IBD	Blood	polyA	Illumina TruSeq RNA sample prep kit	PAXgene Blood RNA kit	Male	38	NA	NA	None
GSM4261011	GSE143507	Blood IBD	IBD	Blood	polyA	Illumina TruSeq RNA sample prep kit	PAXgene Blood RNA kit	Male	43	NA	NA	None
GSM4261012	GSE143507	Blood IBD	IBD	Blood	polyA	Illumina TruSeq RNA sample prep kit	PAXgene Blood RNA kit	Female	35	NA	NA	None
GSM4261013	GSE143507	Blood IBD	IBD	Blood	polyA	Illumina TruSeq RNA sample prep kit	PAXgene Blood RNA kit	Male	44	NA	NA	None
GSM4261014	GSE143507	Blood IBD	IBD	Blood	polyA	Illumina TruSeq RNA sample prep kit	PAXgene Blood RNA kit	Male	60	NA	NA	None
GSM4261015	GSE143507	Blood IBD	IBD	Blood	polyA	Illumina TruSeq RNA sample prep kit	PAXgene Blood RNA kit	Male	46	NA	NA	None
GSM4261016	GSE143507	Blood IBD	IBD	Blood	polyA	Illumina TruSeq RNA sample prep kit	PAXgene Blood RNA kit	Female	30	NA	NA	None
GSM4261017	GSE143507	Blood IBD	IBD	Blood	polyA	Illumina TruSeq RNA sample prep kit	PAXgene Blood RNA kit	Male	33	NA	NA	None
GSM4261018	GSE143507	Blood IBD	IBD	Blood	polyA	Illumina TruSeq RNA sample prep kit	PAXgene Blood RNA kit	Female	35	NA	NA	None
GSM4261019	GSE143507	Blood IBD	IBD	Blood	polyA	Illumina TruSeq RNA sample prep kit	PAXgene Blood RNA kit	Male	32	NA	NA	None
GSM4261020	GSE143507	Blood IBD	IBD	Blood	polyA	Illumina TruSeq RNA sample prep kit	PAXgene Blood RNA kit	Male	39	NA	NA	None
GSM4261021	GSE143507	Blood IBD	IBD	Blood	polyA	Illumina TruSeq RNA sample prep kit	PAXgene Blood RNA kit	Female	53	NA	NA	None
GSM4261022	GSE143507	Blood IBD	IBD	Blood	polyA	Illumina TruSeq RNA sample prep kit	PAXgene Blood RNA kit	Male	51	NA	NA	None
GSM4261023	GSE143507	Blood IBD	IBD	Blood	polyA	Illumina TruSeq RNA sample prep kit	PAXgene Blood RNA kit	Female	30	NA	NA	None
GSM4261024	GSE143507	Blood IBD	IBD	Blood	polyA	Illumina TruSeq RNA sample prep kit	PAXgene Blood RNA kit	Male	35	NA	NA	None
GSM4261025	GSE143507	Blood IBD	IBD	Blood	polyA	Illumina TruSeq RNA sample prep kit	PAXgene Blood RNA kit	Male	33	NA	NA	None
GSM4261026	GSE143507	Blood IBD	IBD	Blood	polyA	Illumina TruSeq RNA sample prep kit	PAXgene Blood RNA kit	Male	35	NA	NA	None
GSM4261027	GSE143507	Blood IBD	IBD	Blood	polyA	Illumina TruSeq RNA sample prep kit	PAXgene Blood RNA kit	Female	44	NA	NA	None
GSM4818000	GSE159034	Blood IBD	IBD	Blood	Total	NEBNext Ultra II Directional RNA Library Prep Kit	PAXgene Blood RNA kit	NA		NA	NA	Infliximab
GSM4818001	GSE159034	Blood IBD	IBD	Blood	Total	NEBNext Ultra II Directional RNA Library Prep Kit	PAXgene Blood RNA kit	NA		NA	NA	Infliximab
GSM4818002	GSE159034	Blood IBD	IBD	Blood	Total	NEBNext Ultra II Directional RNA Library Prep Kit	PAXgene Blood RNA kit	NA		NA	NA	Adalimumab
GSM4818003	GSE159034	Blood IBD	IBD	Blood	Total	NEBNext Ultra II Directional RNA Library Prep Kit	PAXgene Blood RNA kit	NA		NA	NA	Adalimumab
GSM4818004	GSE159034	Blood IBD	IBD	Blood	Total	NEBNext Ultra II Directional RNA Library Prep Kit	PAXgene Blood RNA kit	NA		NA	NA	Infliximab
GSM4818005	GSE159034	Blood IBD	IBD	Blood	Total	NEBNext Ultra II Directional RNA Library Prep Kit	PAXgene Blood RNA kit	NA		NA	NA	Infliximab
GSM4818006	GSE159034	Blood IBD	IBD	Blood	Total	NEBNext Ultra II Directional RNA Library Prep Kit	PAXgene Blood RNA kit	NA		NA	NA	Infliximab

GSM4818007	GSE159034	Blood IBD	IBD	Blood	Total	NEBNext Ultra II Directional RNA Library Prep Kit	PAXgene Blood RNA kit	NA		NA	NA	Infliximab
GSM4818008	GSE159034	Blood IBD	IBD	Blood	Total	NEBNext Ultra II Directional RNA Library Prep Kit	PAXgene Blood RNA kit	NA		NA	NA	Adalimumab
GSM4818009	GSE159034	Blood IBD	IBD	Blood	Total	NEBNext Ultra II Directional RNA Library Prep Kit	PAXgene Blood RNA kit	NA		NA	NA	Adalimumab
GSM4818010	GSE159034	Blood IBD	IBD	Blood	Total	NEBNext Ultra II Directional RNA Library Prep Kit	PAXgene Blood RNA kit	NA		NA	NA	Infliximab
GSM4818011	GSE159034	Blood IBD	IBD	Blood	Total	NEBNext Ultra II Directional RNA Library Prep Kit	PAXgene Blood RNA kit	NA		NA	NA	Infliximab
GSM4818012	GSE159034	Blood IBD	IBD	Blood	Total	NEBNext Ultra II Directional RNA Library Prep Kit	PAXgene Blood RNA kit	NA		NA	NA	Infliximab
GSM4818013	GSE159034	Blood IBD	IBD	Blood	Total	NEBNext Ultra II Directional RNA Library Prep Kit	PAXgene Blood RNA kit	NA		NA	NA	Infliximab
GSM4818014	GSE159034	Blood IBD	IBD	Blood	Total	NEBNext Ultra II Directional RNA Library Prep Kit	PAXgene Blood RNA kit	NA		NA	NA	Infliximab
GSM4818015	GSE159034	Blood IBD	IBD	Blood	Total	NEBNext Ultra II Directional RNA Library Prep Kit	PAXgene Blood RNA kit	NA		NA	NA	Infliximab
GSM4818016	GSE159034	Blood IBD	IBD	Blood	Total	NEBNext Ultra II Directional RNA Library Prep Kit	PAXgene Blood RNA kit	NA		NA	NA	Infliximab
GSM4818017	GSE159034	Blood IBD	IBD	Blood	Total	NEBNext Ultra II Directional RNA Library Prep Kit	PAXgene Blood RNA kit	NA		NA	NA	Infliximab
GSM4818018	GSE159034	Blood IBD	IBD	Blood	Total	NEBNext Ultra II Directional RNA Library Prep Kit	PAXgene Blood RNA kit	NA		NA	NA	Infliximab
GSM4818019	GSE159034	Blood IBD	IBD	Blood	Total	NEBNext Ultra II Directional RNA Library Prep Kit	PAXgene Blood RNA kit	NA		NA	NA	Infliximab
GSM4818020	GSE159034	Blood IBD	IBD	Blood	Total	NEBNext Ultra II Directional RNA Library Prep Kit	PAXgene Blood RNA kit	NA		NA	NA	Adalimumab
GSM4818021	GSE159034	Blood IBD	IBD	Blood	Total	NEBNext Ultra II Directional RNA Library Prep Kit	PAXgene Blood RNA kit	NA		NA	NA	Adalimumab
GSM4818022	GSE159034	Blood IBD	IBD	Blood	Total	NEBNext Ultra II Directional RNA Library Prep Kit	PAXgene Blood RNA kit	NA		NA	NA	Adalimumab
GSM4818023	GSE159034	Blood IBD	IBD	Blood	Total	NEBNext Ultra II Directional RNA Library Prep Kit	PAXgene Blood RNA kit	NA		NA	NA	Adalimumab
GSM3056394	GSE112057	Blood IBD	IBD	Blood	polyA	Illumina TruSeq RNA sample prep kit	Tempus Blood RNA Tubes	Female		NA	NA	NA
GSM3056395	GSE112057	Blood IBD	IBD	Blood	polyA	Illumina TruSeq RNA sample prep kit	Tempus Blood RNA Tubes	Male		NA	NA	NA
GSM3056396	GSE112057	Blood IBD	IBD	Blood	polyA	Illumina TruSeq RNA sample prep kit	Tempus Blood RNA Tubes	Female		NA	NA	NA
GSM3056397	GSE112057	Blood IBD	IBD	Blood	polyA	Illumina TruSeq RNA sample prep kit	Tempus Blood RNA Tubes	Male		NA	NA	NA

GSM3056398	GSE112057	Blood IBD	IBD	Blood	polyA	Illumina TruSeq RNA sample prep kit	Tempus Blood RNA Tubes	Male		NA	NA	NA
GSM3056399	GSE112057	Blood IBD	IBD	Blood	polyA	Illumina TruSeq RNA sample prep kit	Tempus Blood RNA Tubes	Male		NA	NA	NA
GSM3056400	GSE112057	Blood IBD	IBD	Blood	polyA	Illumina TruSeq RNA sample prep kit	Tempus Blood RNA Tubes	Male		NA	NA	NA
GSM3056401	GSE112057	Blood Control	Control	Blood	polyA	Illumina TruSeq RNA sample prep kit	Tempus Blood RNA Tubes	Female		NA	NA	NA
GSM3056402	GSE112057	Blood Control	Control	Blood	polyA	Illumina TruSeq RNA sample prep kit	Tempus Blood RNA Tubes	Male		NA	NA	NA
GSM3056403	GSE112057	Blood Control	Control	Blood	polyA	Illumina TruSeq RNA sample prep kit	Tempus Blood RNA Tubes	Female		NA	NA	NA
GSM3056404	GSE112057	Blood Control	Control	Blood	polyA	Illumina TruSeq RNA sample prep kit	Tempus Blood RNA Tubes	Female		NA	NA	NA
GSM3056405	GSE112057	Blood Control	Control	Blood	polyA	Illumina TruSeq RNA sample prep kit	Tempus Blood RNA Tubes	Female		NA	NA	NA
GSM3056406	GSE112057	Blood Control	Control	Blood	polyA	Illumina TruSeq RNA sample prep kit	Tempus Blood RNA Tubes	Female		NA	NA	NA
GSM3056407	GSE112057	Blood Control	Control	Blood	polyA	Illumina TruSeq RNA sample prep kit	Tempus Blood RNA Tubes	Female		NA	NA	NA
GSM3056408	GSE112057	Blood Control	Control	Blood	polyA	Illumina TruSeq RNA sample prep kit	Tempus Blood RNA Tubes	Female		NA	NA	NA
GSM3056409	GSE112057	Blood Control	Control	Blood	polyA	Illumina TruSeq RNA sample prep kit	Tempus Blood RNA Tubes	Female		NA	NA	NA
GSM3056410	GSE112057	Blood Control	Control	Blood	polyA	Illumina TruSeq RNA sample prep kit	Tempus Blood RNA Tubes	Female		NA	NA	NA
GSM3056411	GSE112057	Blood IBD	IBD	Blood	polyA	Illumina TruSeq RNA sample prep kit	Tempus Blood RNA Tubes	Female		NA	NA	NA
GSM3056412	GSE112057	Blood IBD	IBD	Blood	polyA	Illumina TruSeq RNA sample prep kit	Tempus Blood RNA Tubes	Female		NA	NA	NA
GSM3056413	GSE112057	Blood IBD	IBD	Blood	polyA	Illumina TruSeq RNA sample prep kit	Tempus Blood RNA Tubes	Male		NA	NA	NA
GSM3056414	GSE112057	Blood IBD	IBD	Blood	polyA	Illumina TruSeq RNA sample prep kit	Tempus Blood RNA Tubes	Male		NA	NA	NA
GSM3056415	GSE112057	Blood Control	Control	Blood	polyA	Illumina TruSeq RNA sample prep kit	Tempus Blood RNA Tubes	Female		NA	NA	NA
GSM3056416	GSE112057	Blood Control	Control	Blood	polyA	Illumina TruSeq RNA sample prep kit	Tempus Blood RNA Tubes	Female		NA	NA	NA
GSM3056417	GSE112057	Blood Control	Control	Blood	polyA	Illumina TruSeq RNA sample prep kit	Tempus Blood RNA Tubes	Male		NA	NA	NA
GSM3056418	GSE112057	Blood Control	Control	Blood	polyA	Illumina TruSeq RNA sample prep kit	Tempus Blood RNA Tubes	Female		NA	NA	NA
GSM3056419	GSE112057	Blood Control	Control	Blood	polyA	Illumina TruSeq RNA sample prep kit	Tempus Blood RNA Tubes	Female		NA	NA	NA
GSM3056420	GSE112057	Blood Control	Control	Blood	polyA	Illumina TruSeq RNA sample prep kit	Tempus Blood RNA Tubes	Female		NA	NA	NA
GSM3056421	GSE112057	Blood Control	Control	Blood	polyA	Illumina TruSeq RNA sample prep kit	Tempus Blood RNA Tubes	Male		NA	NA	NA
GSM3056422	GSE112057	Blood Control	Control	Blood	polyA	Illumina TruSeq RNA sample prep kit	Tempus Blood RNA Tubes	Female		NA	NA	NA
GSM3056423	GSE112057	Blood IBD	IBD	Blood	polyA	Illumina TruSeq RNA sample prep kit	Tempus Blood RNA Tubes	Male		NA	NA	NA
GSM3056424	GSE112057	Blood IBD	IBD	Blood	polyA	Illumina TruSeq RNA sample prep kit	Tempus Blood RNA Tubes	Female		NA	NA	NA
GSM3056425	GSE112057	Blood IBD	IBD	Blood	polyA	Illumina TruSeq RNA sample prep kit	Tempus Blood RNA Tubes	Male		NA	NA	NA
GSM3056426	GSE112057	Blood IBD	IBD	Blood	polyA	Illumina TruSeq RNA sample prep kit	Tempus Blood RNA Tubes	Male		NA	NA	NA

GSM3056427	GSE112057	Blood IBD	IBD	Blood	polyA	Illumina TruSeq RNA sample prep kit	Tempus Blood RNA Tubes	Male		NA	NA	NA
GSM3056428	GSE112057	Blood IBD	IBD	Blood	polyA	Illumina TruSeq RNA sample prep kit	Tempus Blood RNA Tubes	Female		NA	NA	NA
GSM3056429	GSE112057	Blood IBD	IBD	Blood	polyA	Illumina TruSeq RNA sample prep kit	Tempus Blood RNA Tubes	Male		NA	NA	NA
GSM3056430	GSE112057	Blood IBD	IBD	Blood	polyA	Illumina TruSeq RNA sample prep kit	Tempus Blood RNA Tubes	Male		NA	NA	NA
GSM3056431	GSE112057	Blood IBD	IBD	Blood	polyA	Illumina TruSeq RNA sample prep kit	Tempus Blood RNA Tubes	Female		NA	NA	NA
GSM3056432	GSE112057	Blood IBD	IBD	Blood	polyA	Illumina TruSeq RNA sample prep kit	Tempus Blood RNA Tubes	Female		NA	NA	NA
GSM3056433	GSE112057	Blood IBD	IBD	Blood	polyA	Illumina TruSeq RNA sample prep kit	Tempus Blood RNA Tubes	Male		NA	NA	NA
GSM3056434	GSE112057	Blood IBD	IBD	Blood	polyA	Illumina TruSeq RNA sample prep kit	Tempus Blood RNA Tubes	Female		NA	NA	NA
GSM3056435	GSE112057	Blood IBD	IBD	Blood	polyA	Illumina TruSeq RNA sample prep kit	Tempus Blood RNA Tubes	Male		NA	NA	NA
GSM3056436	GSE112057	Blood IBD	IBD	Blood	polyA	Illumina TruSeq RNA sample prep kit	Tempus Blood RNA Tubes	Male		NA	NA	NA
GSM3056437	GSE112057	Blood Control	Control	Blood	polyA	Illumina TruSeq RNA sample prep kit	Tempus Blood RNA Tubes	Female		NA	NA	NA
GSM3056438	GSE112057	Blood Control	Control	Blood	polyA	Illumina TruSeq RNA sample prep kit	Tempus Blood RNA Tubes	Female		NA	NA	NA
GSM3056439	GSE112057	Blood IBD	IBD	Blood	polyA	Illumina TruSeq RNA sample prep kit	Tempus Blood RNA Tubes	Male		NA	NA	NA
GSM3056440	GSE112057	Blood IBD	IBD	Blood	polyA	Illumina TruSeq RNA sample prep kit	Tempus Blood RNA Tubes	Male		NA	NA	NA
GSM3056441	GSE112057	Blood IBD	IBD	Blood	polyA	Illumina TruSeq RNA sample prep kit	Tempus Blood RNA Tubes	Male		NA	NA	NA
GSM3056442	GSE112057	Blood IBD	IBD	Blood	polyA	Illumina TruSeq RNA sample prep kit	Tempus Blood RNA Tubes	Female		NA	NA	NA
GSM3056443	GSE112057	Blood IBD	IBD	Blood	polyA	Illumina TruSeq RNA sample prep kit	Tempus Blood RNA Tubes	Female		NA	NA	NA
GSM3056444	GSE112057	Blood IBD	IBD	Blood	polyA	Illumina TruSeq RNA sample prep kit	Tempus Blood RNA Tubes	Male		NA	NA	NA
GSM3056445	GSE112057	Blood IBD	IBD	Blood	polyA	Illumina TruSeq RNA sample prep kit	Tempus Blood RNA Tubes	Female		NA	NA	NA
GSM3056446	GSE112057	Blood Control	Control	Blood	polyA	Illumina TruSeq RNA sample prep kit	Tempus Blood RNA Tubes	Female		NA	NA	NA
GSM3056447	GSE112057	Blood Control	Control	Blood	polyA	Illumina TruSeq RNA sample prep kit	Tempus Blood RNA Tubes	Female		NA	NA	NA
GSM3056448	GSE112057	Blood Control	Control	Blood	polyA	Illumina TruSeq RNA sample prep kit	Tempus Blood RNA Tubes	Female		NA	NA	NA
GSM3056449	GSE112057	Blood Control	Control	Blood	polyA	Illumina TruSeq RNA sample prep kit	Tempus Blood RNA Tubes	Female		NA	NA	NA
GSM3056450	GSE112057	Blood Control	Control	Blood	polyA	Illumina TruSeq RNA sample prep kit	Tempus Blood RNA Tubes	Female		NA	NA	NA
GSM3056451	GSE112057	Blood Control	Control	Blood	polyA	Illumina TruSeq RNA sample prep kit	Tempus Blood RNA Tubes	Male		NA	NA	NA
GSM3056452	GSE112057	Blood Control	Control	Blood	polyA	Illumina TruSeq RNA sample prep kit	Tempus Blood RNA Tubes	Male		NA	NA	NA
GSM3056453	GSE112057	Blood Control	Control	Blood	polyA	Illumina TruSeq RNA sample prep kit	Tempus Blood RNA Tubes	Female		NA	NA	NA
GSM3056454	GSE112057	Blood Control	Control	Blood	polyA	Illumina TruSeq RNA sample prep kit	Tempus Blood RNA Tubes	Female		NA	NA	NA
GSM3056455	GSE112057	Blood Control	Control	Blood	polyA	Illumina TruSeq RNA sample prep kit	Tempus Blood RNA Tubes	Female		NA	NA	NA

GSM3056456	GSE112057	Blood Control	Control	Blood	polyA	Illumina TruSeq RNA sample prep kit	Tempus Blood RNA Tubes	Female		NA	NA	NA
GSM3056457	GSE112057	Blood Control	Control	Blood	polyA	Illumina TruSeq RNA sample prep kit	Tempus Blood RNA Tubes	Female		NA	NA	NA
GSM3056458	GSE112057	Blood IBD	IBD	Blood	polyA	Illumina TruSeq RNA sample prep kit	Tempus Blood RNA Tubes	Male		NA	NA	NA
GSM3056459	GSE112057	Blood IBD	IBD	Blood	polyA	Illumina TruSeq RNA sample prep kit	Tempus Blood RNA Tubes	Male		NA	NA	NA
GSM3056460	GSE112057	Blood IBD	IBD	Blood	polyA	Illumina TruSeq RNA sample prep kit	Tempus Blood RNA Tubes	Female		NA	NA	NA
GSM3056461	GSE112057	Blood IBD	IBD	Blood	polyA	Illumina TruSeq RNA sample prep kit	Tempus Blood RNA Tubes	Male		NA	NA	NA
GSM3056462	GSE112057	Blood IBD	IBD	Blood	polyA	Illumina TruSeq RNA sample prep kit	Tempus Blood RNA Tubes	Female		NA	NA	NA
GSM3056463	GSE112057	Blood IBD	IBD	Blood	polyA	Illumina TruSeq RNA sample prep kit	Tempus Blood RNA Tubes	Male		NA	NA	NA
GSM3056464	GSE112057	Blood IBD	IBD	Blood	polyA	Illumina TruSeq RNA sample prep kit	Tempus Blood RNA Tubes	Female		NA	NA	NA
GSM3056465	GSE112057	Blood Control	Control	Blood	polyA	Illumina TruSeq RNA sample prep kit	Tempus Blood RNA Tubes	Female		NA	NA	NA
GSM3056466	GSE112057	Blood Control	Control	Blood	polyA	Illumina TruSeq RNA sample prep kit	Tempus Blood RNA Tubes	Female		NA	NA	NA
GSM3056467	GSE112057	Blood Control	Control	Blood	polyA	Illumina TruSeq RNA sample prep kit	Tempus Blood RNA Tubes	Male		NA	NA	NA
GSM3056468	GSE112057	Blood Control	Control	Blood	polyA	Illumina TruSeq RNA sample prep kit	Tempus Blood RNA Tubes	Male		NA	NA	NA
GSM3056469	GSE112057	Blood Control	Control	Blood	polyA	Illumina TruSeq RNA sample prep kit	Tempus Blood RNA Tubes	Male		NA	NA	NA
GSM3056470	GSE112057	Blood Control	Control	Blood	polyA	Illumina TruSeq RNA sample prep kit	Tempus Blood RNA Tubes	Female		NA	NA	NA
GSM3056471	GSE112057	Blood Control	Control	Blood	polyA	Illumina TruSeq RNA sample prep kit	Tempus Blood RNA Tubes	Female		NA	NA	NA
GSM3056472	GSE112057	Blood Control	Control	Blood	polyA	Illumina TruSeq RNA sample prep kit	Tempus Blood RNA Tubes	Male		NA	NA	NA
GSM3056473	GSE112057	Blood Control	Control	Blood	polyA	Illumina TruSeq RNA sample prep kit	Tempus Blood RNA Tubes	Female		NA	NA	NA
GSM3056474	GSE112057	Blood IBD	IBD	Blood	polyA	Illumina TruSeq RNA sample prep kit	Tempus Blood RNA Tubes	Female		NA	NA	NA
GSM3056475	GSE112057	Blood IBD	IBD	Blood	polyA	Illumina TruSeq RNA sample prep kit	Tempus Blood RNA Tubes	Male		NA	NA	NA
GSM3056476	GSE112057	Blood IBD	IBD	Blood	polyA	Illumina TruSeq RNA sample prep kit	Tempus Blood RNA Tubes	Female		NA	NA	NA
GSM3056477	GSE112057	Blood IBD	IBD	Blood	polyA	Illumina TruSeq RNA sample prep kit	Tempus Blood RNA Tubes	Female		NA	NA	NA
GSM3056478	GSE112057	Blood Control	Control	Blood	polyA	Illumina TruSeq RNA sample prep kit	Tempus Blood RNA Tubes	Female		NA	NA	NA
GSM3056479	GSE112057	Blood Control	Control	Blood	polyA	Illumina TruSeq RNA sample prep kit	Tempus Blood RNA Tubes	Female		NA	NA	NA
GSM3056480	GSE112057	Blood Control	Control	Blood	polyA	Illumina TruSeq RNA sample prep kit	Tempus Blood RNA Tubes	Female		NA	NA	NA
GSM3056481	GSE112057	Blood Control	Control	Blood	polyA	Illumina TruSeq RNA sample prep kit	Tempus Blood RNA Tubes	Male		NA	NA	NA
GSM3056482	GSE112057	Blood Control	Control	Blood	polyA	Illumina TruSeq RNA sample prep kit	Tempus Blood RNA Tubes	Female		NA	NA	NA
GSM3056483	GSE112057	Blood Control	Control	Blood	polyA	Illumina TruSeq RNA sample prep kit	Tempus Blood RNA Tubes	Female		NA	NA	NA
GSM3056484	GSE112057	Blood Control	Control	Blood	polyA	Illumina TruSeq RNA sample prep kit	Tempus Blood RNA Tubes	Female		NA	NA	NA

GSM3056485	GSE112057	Blood Control	Control	Blood	polyA	Illumina TruSeq RNA sample prep kit	Tempus Blood RNA Tubes	Female		NA	NA	NA
GSM3056486	GSE112057	Blood Control	Control	Blood	polyA	Illumina TruSeq RNA sample prep kit	Tempus Blood RNA Tubes	Male		NA	NA	NA
GSM3056487	GSE112057	Blood Control	Control	Blood	polyA	Illumina TruSeq RNA sample prep kit	Tempus Blood RNA Tubes	Female		NA	NA	NA
GSM3056488	GSE112057	Blood Control	Control	Blood	polyA	Illumina TruSeq RNA sample prep kit	Tempus Blood RNA Tubes	Male		NA	NA	NA
GSM3056489	GSE112057	Blood Control	Control	Blood	polyA	Illumina TruSeq RNA sample prep kit	Tempus Blood RNA Tubes	Male		NA	NA	NA
GSM3056490	GSE112057	Blood IBD	IBD	Blood	polyA	Illumina TruSeq RNA sample prep kit	Tempus Blood RNA Tubes	Male		NA	NA	NA
GSM3056491	GSE112057	Blood IBD	IBD	Blood	polyA	Illumina TruSeq RNA sample prep kit	Tempus Blood RNA Tubes	Female		NA	NA	NA
GSM3056492	GSE112057	Blood IBD	IBD	Blood	polyA	Illumina TruSeq RNA sample prep kit	Tempus Blood RNA Tubes	Female		NA	NA	NA
GSM3056493	GSE112057	Blood IBD	IBD	Blood	polyA	Illumina TruSeq RNA sample prep kit	Tempus Blood RNA Tubes	Male		NA	NA	NA
GSM3056494	GSE112057	Blood Control	Control	Blood	polyA	Illumina TruSeq RNA sample prep kit	Tempus Blood RNA Tubes	Female		NA	NA	NA
GSM3056495	GSE112057	Blood Control	Control	Blood	polyA	Illumina TruSeq RNA sample prep kit	Tempus Blood RNA Tubes	Male		NA	NA	NA
GSM3056496	GSE112057	Blood Control	Control	Blood	polyA	Illumina TruSeq RNA sample prep kit	Tempus Blood RNA Tubes	Female		NA	NA	NA
GSM3056497	GSE112057	Blood Control	Control	Blood	polyA	Illumina TruSeq RNA sample prep kit	Tempus Blood RNA Tubes	Female		NA	NA	NA
GSM3056498	GSE112057	Blood Control	Control	Blood	polyA	Illumina TruSeq RNA sample prep kit	Tempus Blood RNA Tubes	Male		NA	NA	NA
GSM3056499	GSE112057	Blood Control	Control	Blood	polyA	Illumina TruSeq RNA sample prep kit	Tempus Blood RNA Tubes	Male		NA	NA	NA
GSM3056500	GSE112057	Blood Control	Control	Blood	polyA	Illumina TruSeq RNA sample prep kit	Tempus Blood RNA Tubes	Male		NA	NA	NA
GSM3056501	GSE112057	Blood Control	Control	Blood	polyA	Illumina TruSeq RNA sample prep kit	Tempus Blood RNA Tubes	Female		NA	NA	NA
GSM3056502	GSE112057	Blood Control	Control	Blood	polyA	Illumina TruSeq RNA sample prep kit	Tempus Blood RNA Tubes	Male		NA	NA	NA
GSM3056503	GSE112057	Blood Control	Control	Blood	polyA	Illumina TruSeq RNA sample prep kit	Tempus Blood RNA Tubes	Female		NA	NA	NA
GSM3056504	GSE112057	Blood IBD	IBD	Blood	polyA	Illumina TruSeq RNA sample prep kit	Tempus Blood RNA Tubes	Female		NA	NA	NA
GSM3056505	GSE112057	Blood IBD	IBD	Blood	polyA	Illumina TruSeq RNA sample prep kit	Tempus Blood RNA Tubes	Male		NA	NA	NA
GSM3056506	GSE112057	Blood IBD	IBD	Blood	polyA	Illumina TruSeq RNA sample prep kit	Tempus Blood RNA Tubes	Male		NA	NA	NA
GSM3056507	GSE112057	Blood IBD	IBD	Blood	polyA	Illumina TruSeq RNA sample prep kit	Tempus Blood RNA Tubes	Male		NA	NA	NA
GSM3056508	GSE112057	Blood IBD	IBD	Blood	polyA	Illumina TruSeq RNA sample prep kit	Tempus Blood RNA Tubes	Female		NA	NA	NA
GSM3056509	GSE112057	Blood IBD	IBD	Blood	polyA	Illumina TruSeq RNA sample prep kit	Tempus Blood RNA Tubes	Male		NA	NA	NA
GSM3056510	GSE112057	Blood Control	Control	Blood	polyA	Illumina TruSeq RNA sample prep kit	Tempus Blood RNA Tubes	Female		NA	NA	NA
GSM3056511	GSE112057	Blood Control	Control	Blood	polyA	Illumina TruSeq RNA sample prep kit	Tempus Blood RNA Tubes	Male		NA	NA	NA
GSM3056512	GSE112057	Blood Control	Control	Blood	polyA	Illumina TruSeq RNA sample prep kit	Tempus Blood RNA Tubes	Female		NA	NA	NA
GSM3056513	GSE112057	Blood Control	Control	Blood	polyA	Illumina TruSeq RNA sample prep kit	Tempus Blood RNA Tubes	Male		NA	NA	NA

GSM3056514	GSE112057	Blood Control	Control	Blood	polyA	Illumina TruSeq RNA sample prep kit	Tempus Blood RNA Tubes	Female		NA	NA	NA
GSM3056515	GSE112057	Blood Control	Control	Blood	polyA	Illumina TruSeq RNA sample prep kit	Tempus Blood RNA Tubes	Male		NA	NA	NA
GSM3056516	GSE112057	Blood Control	Control	Blood	polyA	Illumina TruSeq RNA sample prep kit	Tempus Blood RNA Tubes	Male		NA	NA	NA
GSM3056517	GSE112057	Blood Control	Control	Blood	polyA	Illumina TruSeq RNA sample prep kit	Tempus Blood RNA Tubes	Female		NA	NA	NA
GSM3056518	GSE112057	Blood Control	Control	Blood	polyA	Illumina TruSeq RNA sample prep kit	Tempus Blood RNA Tubes	Female		NA	NA	NA
GSM3056519	GSE112057	Blood IBD	IBD	Blood	polyA	Illumina TruSeq RNA sample prep kit	Tempus Blood RNA Tubes	Male		NA	NA	NA
GSM3056520	GSE112057	Blood IBD	IBD	Blood	polyA	Illumina TruSeq RNA sample prep kit	Tempus Blood RNA Tubes	Male		NA	NA	NA
GSM3056521	GSE112057	Blood IBD	IBD	Blood	polyA	Illumina TruSeq RNA sample prep kit	Tempus Blood RNA Tubes	Male		NA	NA	NA
GSM3056522	GSE112057	Blood IBD	IBD	Blood	polyA	Illumina TruSeq RNA sample prep kit	Tempus Blood RNA Tubes	Female		NA	NA	NA
GSM3056523	GSE112057	Blood IBD	IBD	Blood	polyA	Illumina TruSeq RNA sample prep kit	Tempus Blood RNA Tubes	Female		NA	NA	NA
GSM3056524	GSE112057	Blood IBD	IBD	Blood	polyA	Illumina TruSeq RNA sample prep kit	Tempus Blood RNA Tubes	Female		NA	NA	NA
GSM3056525	GSE112057	Blood IBD	IBD	Blood	polyA	Illumina TruSeq RNA sample prep kit	Tempus Blood RNA Tubes	Male		NA	NA	NA
GSM3056526	GSE112057	Blood Control	Control	Blood	polyA	Illumina TruSeq RNA sample prep kit	Tempus Blood RNA Tubes	Female		NA	NA	NA
GSM3056527	GSE112057	Blood Control	Control	Blood	polyA	Illumina TruSeq RNA sample prep kit	Tempus Blood RNA Tubes	Male		NA	NA	NA
GSM3056528	GSE112057	Blood Control	Control	Blood	polyA	Illumina TruSeq RNA sample prep kit	Tempus Blood RNA Tubes	Female		NA	NA	NA
GSM3056529	GSE112057	Blood Control	Control	Blood	polyA	Illumina TruSeq RNA sample prep kit	Tempus Blood RNA Tubes	Female		NA	NA	NA
GSM3056530	GSE112057	Blood Control	Control	Blood	polyA	Illumina TruSeq RNA sample prep kit	Tempus Blood RNA Tubes	Male		NA	NA	NA
GSM3056531	GSE112057	Blood Control	Control	Blood	polyA	Illumina TruSeq RNA sample prep kit	Tempus Blood RNA Tubes	Female		NA	NA	NA
GSM3056532	GSE112057	Blood Control	Control	Blood	polyA	Illumina TruSeq RNA sample prep kit	Tempus Blood RNA Tubes	Female		NA	NA	NA
GSM3056533	GSE112057	Blood Control	Control	Blood	polyA	Illumina TruSeq RNA sample prep kit	Tempus Blood RNA Tubes	Female		NA	NA	NA
GSM3056534	GSE112057	Blood Control	Control	Blood	polyA	Illumina TruSeq RNA sample prep kit	Tempus Blood RNA Tubes	Male		NA	NA	NA
GSM3056535	GSE112057	Blood Control	Control	Blood	polyA	Illumina TruSeq RNA sample prep kit	Tempus Blood RNA Tubes	Male		NA	NA	NA
GSM3056536	GSE112057	Blood Control	Control	Blood	polyA	Illumina TruSeq RNA sample prep kit	Tempus Blood RNA Tubes	Female		NA	NA	NA
GSM3056537	GSE112057	Blood IBD	IBD	Blood	polyA	Illumina TruSeq RNA sample prep kit	Tempus Blood RNA Tubes	Male		NA	NA	NA
GSM3056538	GSE112057	Blood IBD	IBD	Blood	polyA	Illumina TruSeq RNA sample prep kit	Tempus Blood RNA Tubes	Male		NA	NA	NA
GSM3056539	GSE112057	Blood IBD	IBD	Blood	polyA	Illumina TruSeq RNA sample prep kit	Tempus Blood RNA Tubes	Male		NA	NA	NA
GSM3056540	GSE112057	Blood IBD	IBD	Blood	polyA	Illumina TruSeq RNA sample prep kit	Tempus Blood RNA Tubes	Female		NA	NA	NA
GSM3056541	GSE112057	Blood IBD	IBD	Blood	polyA	Illumina TruSeq RNA sample prep kit	Tempus Blood RNA Tubes	Female		NA	NA	NA
GSM3056542	GSE112057	Blood IBD	IBD	Blood	polyA	Illumina TruSeq RNA sample prep kit	Tempus Blood RNA Tubes	Male		NA	NA	NA

GSM3056543	GSE112057	Blood IBD	IBD	Blood	polyA	Illumina TruSeq RNA sample prep kit	Tempus Blood RNA Tubes	Female		NA	NA	NA
GSM3056544	GSE112057	Blood Control	Control	Blood	polyA	Illumina TruSeq RNA sample prep kit	Tempus Blood RNA Tubes	Male		NA	NA	NA
GSM3056545	GSE112057	Blood Control	Control	Blood	polyA	Illumina TruSeq RNA sample prep kit	Tempus Blood RNA Tubes	Female		NA	NA	NA
GSM3056546	GSE112057	Blood Control	Control	Blood	polyA	Illumina TruSeq RNA sample prep kit	Tempus Blood RNA Tubes	Female		NA	NA	NA
GSM3056547	GSE112057	Blood Control	Control	Blood	polyA	Illumina TruSeq RNA sample prep kit	Tempus Blood RNA Tubes	Male		NA	NA	NA
GSM3056548	GSE112057	Blood Control	Control	Blood	polyA	Illumina TruSeq RNA sample prep kit	Tempus Blood RNA Tubes	Male		NA	NA	NA
GSM3056549	GSE112057	Blood Control	Control	Blood	polyA	Illumina TruSeq RNA sample prep kit	Tempus Blood RNA Tubes	Female		NA	NA	NA
GSM3056550	GSE112057	Blood Control	Control	Blood	polyA	Illumina TruSeq RNA sample prep kit	Tempus Blood RNA Tubes	Male		NA	NA	NA
GSM3056551	GSE112057	Blood Control	Control	Blood	polyA	Illumina TruSeq RNA sample prep kit	Tempus Blood RNA Tubes	Female		NA	NA	NA
GSM3056552	GSE112057	Blood IBD	IBD	Blood	polyA	Illumina TruSeq RNA sample prep kit	Tempus Blood RNA Tubes	Male		NA	NA	NA
GSM3056553	GSE112057	Blood IBD	IBD	Blood	polyA	Illumina TruSeq RNA sample prep kit	Tempus Blood RNA Tubes	Male		NA	NA	NA
GSM3056554	GSE112057	Blood IBD	IBD	Blood	polyA	Illumina TruSeq RNA sample prep kit	Tempus Blood RNA Tubes	Male		NA	NA	NA
GSM3056555	GSE112057	Blood IBD	IBD	Blood	polyA	Illumina TruSeq RNA sample prep kit	Tempus Blood RNA Tubes	Female		NA	NA	NA
GSM3056556	GSE112057	Blood IBD	IBD	Blood	polyA	Illumina TruSeq RNA sample prep kit	Tempus Blood RNA Tubes	Male		NA	NA	NA
GSM3056557	GSE112057	Blood Control	Control	Blood	polyA	Illumina TruSeq RNA sample prep kit	Tempus Blood RNA Tubes	Male		NA	NA	NA
GSM3056558	GSE112057	Blood Control	Control	Blood	polyA	Illumina TruSeq RNA sample prep kit	Tempus Blood RNA Tubes	Female		NA	NA	NA
GSM3056559	GSE112057	Blood Control	Control	Blood	polyA	Illumina TruSeq RNA sample prep kit	Tempus Blood RNA Tubes	Female		NA	NA	NA
GSM3056560	GSE112057	Blood Control	Control	Blood	polyA	Illumina TruSeq RNA sample prep kit	Tempus Blood RNA Tubes	Male		NA	NA	NA
GSM3056561	GSE112057	Blood Control	Control	Blood	polyA	Illumina TruSeq RNA sample prep kit	Tempus Blood RNA Tubes	Male		NA	NA	NA
GSM3056562	GSE112057	Blood Control	Control	Blood	polyA	Illumina TruSeq RNA sample prep kit	Tempus Blood RNA Tubes	Female		NA	NA	NA
GSM3056563	GSE112057	Blood Control	Control	Blood	polyA	Illumina TruSeq RNA sample prep kit	Tempus Blood RNA Tubes	Male		NA	NA	NA
GSM3056564	GSE112057	Blood Control	Control	Blood	polyA	Illumina TruSeq RNA sample prep kit	Tempus Blood RNA Tubes	Female		NA	NA	NA
GSM3056565	GSE112057	Blood Control	Control	Blood	polyA	Illumina TruSeq RNA sample prep kit	Tempus Blood RNA Tubes	Female		NA	NA	NA
GSM3056566	GSE112057	Blood Control	Control	Blood	polyA	Illumina TruSeq RNA sample prep kit	Tempus Blood RNA Tubes	Female		NA	NA	NA
GSM3056567	GSE112057	Blood IBD	IBD	Blood	polyA	Illumina TruSeq RNA sample prep kit	Tempus Blood RNA Tubes	Male		NA	NA	NA
GSM3056568	GSE112057	Blood IBD	IBD	Blood	polyA	Illumina TruSeq RNA sample prep kit	Tempus Blood RNA Tubes	Female		NA	NA	NA
GSM3056569	GSE112057	Blood Control	Control	Blood	polyA	Illumina TruSeq RNA sample prep kit	Tempus Blood RNA Tubes	NA		NA	NA	NA
GSM3056570	GSE112057	Blood Control	Control	Blood	polyA	Illumina TruSeq RNA sample prep kit	Tempus Blood RNA Tubes	NA		NA	NA	NA
GSM3056571	GSE112057	Blood Control	Control	Blood	polyA	Illumina TruSeq RNA sample prep kit	Tempus Blood RNA Tubes	NA		NA	NA	NA

GSM3056572	GSE112057	Blood Control	Control	Blood	polyA	Illumina TruSeq RNA sample prep kit	Tempus Blood RNA Tubes	NA		NA	NA	NA
GSM3056573	GSE112057	Blood Control	Control	Blood	polyA	Illumina TruSeq RNA sample prep kit	Tempus Blood RNA Tubes	NA		NA	NA	NA
GSM3056574	GSE112057	Blood Control	Control	Blood	polyA	Illumina TruSeq RNA sample prep kit	Tempus Blood RNA Tubes	NA		NA	NA	NA
GSM3056575	GSE112057	Blood Control	Control	Blood	polyA	Illumina TruSeq RNA sample prep kit	Tempus Blood RNA Tubes	NA		NA	NA	NA
GSM3056576	GSE112057	Blood Control	Control	Blood	polyA	Illumina TruSeq RNA sample prep kit	Tempus Blood RNA Tubes	NA		NA	NA	NA
GSM3056577	GSE112057	Blood Control	Control	Blood	polyA	Illumina TruSeq RNA sample prep kit	Tempus Blood RNA Tubes	NA		NA	NA	NA
GSM3056578	GSE112057	Blood Control	Control	Blood	polyA	Illumina TruSeq RNA sample prep kit	Tempus Blood RNA Tubes	NA		NA	NA	NA
GSM3056579	GSE112057	Blood Control	Control	Blood	polyA	Illumina TruSeq RNA sample prep kit	Tempus Blood RNA Tubes	NA		NA	NA	NA
GSM3056580	GSE112057	Blood Control	Control	Blood	polyA	Illumina TruSeq RNA sample prep kit	Tempus Blood RNA Tubes	NA		NA	NA	NA
GSM3056581	GSE112057	Blood Control	Control	Blood	polyA	Illumina TruSeq RNA sample prep kit	Tempus Blood RNA Tubes	Male		NA	NA	NA
GSM3056582	GSE112057	Blood Control	Control	Blood	polyA	Illumina TruSeq RNA sample prep kit	Tempus Blood RNA Tubes	Female		NA	NA	NA
GSM3056583	GSE112057	Blood Control	Control	Blood	polyA	Illumina TruSeq RNA sample prep kit	Tempus Blood RNA Tubes	Female		NA	NA	NA
GSM3056584	GSE112057	Blood Control	Control	Blood	polyA	Illumina TruSeq RNA sample prep kit	Tempus Blood RNA Tubes	Female		NA	NA	NA
GSM3056585	GSE112057	Blood Control	Control	Blood	polyA	Illumina TruSeq RNA sample prep kit	Tempus Blood RNA Tubes	Female		NA	NA	NA
GSM3056586	GSE112057	Blood Control	Control	Blood	polyA	Illumina TruSeq RNA sample prep kit	Tempus Blood RNA Tubes	Female		NA	NA	NA
GSM3056587	GSE112057	Blood Control	Control	Blood	polyA	Illumina TruSeq RNA sample prep kit	Tempus Blood RNA Tubes	Female		NA	NA	NA
GSM3056588	GSE112057	Blood Control	Control	Blood	polyA	Illumina TruSeq RNA sample prep kit	Tempus Blood RNA Tubes	Female		NA	NA	NA
GSM3056589	GSE112057	Blood Control	Control	Blood	polyA	Illumina TruSeq RNA sample prep kit	Tempus Blood RNA Tubes	Female		NA	NA	NA
GSM3056590	GSE112057	Blood Control	Control	Blood	polyA	Illumina TruSeq RNA sample prep kit	Tempus Blood RNA Tubes	Male		NA	NA	NA
GSM3056591	GSE112057	Blood Control	Control	Blood	polyA	Illumina TruSeq RNA sample prep kit	Tempus Blood RNA Tubes	Female		NA	NA	NA
GSM3056592	GSE112057	Blood Control	Control	Blood	polyA	Illumina TruSeq RNA sample prep kit	Tempus Blood RNA Tubes	Female		NA	NA	NA
GSM3056593	GSE112057	Blood Control	Control	Blood	polyA	Illumina TruSeq RNA sample prep kit	Tempus Blood RNA Tubes	Female		NA	NA	NA
GSM3056594	GSE112057	Blood Control	Control	Blood	polyA	Illumina TruSeq RNA sample prep kit	Tempus Blood RNA Tubes	Female		NA	NA	NA
GSM3056595	GSE112057	Blood IBD	IBD	Blood	polyA	Illumina TruSeq RNA sample prep kit	Tempus Blood RNA Tubes	Female		NA	NA	NA
GSM1701327	GSE69446	Blood IBD	IBD	Blood	Total	Illumina TruSeq RNA sample prep kit	NucleoSpin miRNA kit	NA		NA	Monocytes	MDP-stimulated
GSM1701328	GSE69446	Blood IBD	IBD	Blood	Total	Illumina TruSeq RNA sample prep kit	NucleoSpin miRNA kit	NA		NA	Monocytes	MDP-stimulated
GSM1701329	GSE69446	Blood IBD	IBD	Blood	Total	Illumina TruSeq RNA sample prep kit	NucleoSpin miRNA kit	NA		NA	Monocytes	MDP-stimulated
GSM1701330	GSE69446	Blood IBD	IBD	Blood	Total	Illumina TruSeq RNA sample prep kit	NucleoSpin miRNA kit	NA		NA	Monocytes	Unstimulated
GSM1701331	GSE69446	Blood IBD	IBD	Blood	Total	Illumina TruSeq RNA sample prep kit	NucleoSpin miRNA kit	NA		NA	Monocytes	Unstimulated

GSM1701332	GSE69446	Blood IBD	IBD	Blood	Total	Illumina TruSeq RNA sample prep kit	NucleoSpin miRNA kit	NA		NA	Monocytes	Unstimulated
GSM1701333	GSE69446	Blood IBD	IBD	Blood	Total	Illumina TruSeq RNA sample prep kit	NucleoSpin miRNA kit	NA		NA	Monocytes	MDP-stimulated
GSM1701334	GSE69446	Blood IBD	IBD	Blood	Total	Illumina TruSeq RNA sample prep kit	NucleoSpin miRNA kit	NA		NA	Monocytes	MDP-stimulated
GSM1701335	GSE69446	Blood IBD	IBD	Blood	Total	Illumina TruSeq RNA sample prep kit	NucleoSpin miRNA kit	NA		NA	Monocytes	Unstimulated
GSM1701336	GSE69446	Blood IBD	IBD	Blood	Total	Illumina TruSeq RNA sample prep kit	NucleoSpin miRNA kit	NA		NA	Monocytes	Unstimulated
GSM1701337	GSE69446	Blood Control	Control	Blood	Total	Illumina TruSeq RNA sample prep kit	NucleoSpin miRNA kit	NA		NA	Monocytes	MDP-stimulated
GSM1701338	GSE69446	Blood Control	Control	Blood	Total	Illumina TruSeq RNA sample prep kit	NucleoSpin miRNA kit	NA		NA	Monocytes	MDP-stimulated
GSM1701339	GSE69446	Blood Control	Control	Blood	Total	Illumina TruSeq RNA sample prep kit	NucleoSpin miRNA kit	NA		NA	Monocytes	Unstimulated
GSM1701340	GSE69446	Blood Control	Control	Blood	Total	Illumina TruSeq RNA sample prep kit	NucleoSpin miRNA kit	NA		NA	Monocytes	Unstimulated
GSM1701341	GSE69446	Blood IBD	IBD	Blood	Total	Illumina TruSeq RNA sample prep kit	NucleoSpin miRNA kit	NA		NA	Monocytes	MDP-stimulated
GSM1701342	GSE69446	Blood IBD	IBD	Blood	Total	Illumina TruSeq RNA sample prep kit	NucleoSpin miRNA kit	NA		NA	Monocytes	MDP-stimulated
GSM1701343	GSE69446	Blood IBD	IBD	Blood	Total	Illumina TruSeq RNA sample prep kit	NucleoSpin miRNA kit	NA		NA	Monocytes	MDP-stimulated
GSM1701344	GSE69446	Blood IBD	IBD	Blood	Total	Illumina TruSeq RNA sample prep kit	NucleoSpin miRNA kit	NA		NA	Monocytes	Unstimulated
GSM1701345	GSE69446	Blood IBD	IBD	Blood	Total	Illumina TruSeq RNA sample prep kit	NucleoSpin miRNA kit	NA		NA	Monocytes	Unstimulated
GSM1701346	GSE69446	Blood IBD	IBD	Blood	Total	Illumina TruSeq RNA sample prep kit	NucleoSpin miRNA kit	NA		NA	Monocytes	Unstimulated
GSM1701347	GSE69446	Blood IBD	IBD	Blood	Total	Illumina TruSeq RNA sample prep kit	NucleoSpin miRNA kit	NA		NA	Monocytes	MDP-stimulated
GSM1701348	GSE69446	Blood IBD	IBD	Blood	Total	Illumina TruSeq RNA sample prep kit	NucleoSpin miRNA kit	NA		NA	Monocytes	MDP-stimulated
GSM1701349	GSE69446	Blood IBD	IBD	Blood	Total	Illumina TruSeq RNA sample prep kit	NucleoSpin miRNA kit	NA		NA	Monocytes	Unstimulated
GSM1701350	GSE69446	Blood IBD	IBD	Blood	Total	Illumina TruSeq RNA sample prep kit	NucleoSpin miRNA kit	NA		NA	Monocytes	Unstimulated
GSM1701351	GSE69446	Blood Control	Control	Blood	Total	Illumina TruSeq RNA sample prep kit	NucleoSpin miRNA kit	NA		NA	Monocytes	MDP-stimulated
GSM1701352	GSE69446	Blood Control	Control	Blood	Total	Illumina TruSeq RNA sample prep kit	NucleoSpin miRNA kit	NA		NA	Monocytes	MDP-stimulated
GSM1701353	GSE69446	Blood Control	Control	Blood	Total	Illumina TruSeq RNA sample prep kit	NucleoSpin miRNA kit	NA		NA	Monocytes	Unstimulated
GSM1701354	GSE69446	Blood Control	Control	Blood	Total	Illumina TruSeq RNA sample prep kit	NucleoSpin miRNA kit	NA		NA	Monocytes	Unstimulated
GSM2430372	GSE92472	Blood IBD	IBD	Blood	polyA	Illumina TruSeq Stranded mRNA kit	PAXgene Blood RNA kit	NA		NA	NA	NA
GSM2430373	GSE92472	Blood IBD	IBD	Blood	polyA	Illumina TruSeq Stranded mRNA kit	PAXgene Blood RNA kit	NA		NA	NA	NA
GSM2430374	GSE92472	Blood IBD	IBD	Blood	polyA	Illumina TruSeq Stranded mRNA kit	PAXgene Blood RNA kit	NA		NA	NA	NA
GSM2430375	GSE92472	Blood Control	Control	Blood	polyA	Illumina TruSeq Stranded mRNA kit	PAXgene Blood RNA kit	NA		NA	NA	NA
GSM2430376	GSE92472	Blood Control	Control	Blood	polyA	Illumina TruSeq Stranded mRNA kit	PAXgene Blood RNA kit	NA		NA	NA	NA
GSM2430377	GSE92472	Blood Control	Control	Blood	polyA	Illumina TruSeq Stranded mRNA kit	PAXgene Blood RNA kit	NA		NA	NA	NA

GSM2430378	GSE92472	Blood Control	Control	Blood	polyA	Illumina TruSeq Stranded mRNA kit	PAXgene Blood RNA kit	NA		NA	NA	NA
GSM2430379	GSE92472	Blood Control	Control	Blood	polyA	Illumina TruSeq Stranded mRNA kit	PAXgene Blood RNA kit	NA		NA	NA	NA
GSM2430380	GSE92472	Blood Control	Control	Blood	polyA	Illumina TruSeq Stranded mRNA kit	PAXgene Blood RNA kit	NA		NA	NA	NA
GSM2430381	GSE92472	Blood Control	Control	Blood	polyA	Illumina TruSeq Stranded mRNA kit	PAXgene Blood RNA kit	NA		NA	NA	NA
GSM2430382	GSE92472	Blood Control	Control	Blood	polyA	Illumina TruSeq Stranded mRNA kit	PAXgene Blood RNA kit	NA		NA	NA	NA
GSM2430383	GSE92472	Blood Control	Control	Blood	polyA	Illumina TruSeq Stranded mRNA kit	PAXgene Blood RNA kit	NA		NA	NA	NA
GSM2430384	GSE92472	Blood Control	Control	Blood	polyA	Illumina TruSeq Stranded mRNA kit	PAXgene Blood RNA kit	NA		NA	NA	NA
GSM2430385	GSE92472	Blood Control	Control	Blood	polyA	Illumina TruSeq Stranded mRNA kit	PAXgene Blood RNA kit	NA		NA	NA	NA
GSM2430386	GSE92472	Blood Control	Control	Blood	polyA	Illumina TruSeq Stranded mRNA kit	PAXgene Blood RNA kit	NA		NA	NA	NA
GSM3496511	GSE123141	Blood IBD	IBD	Blood	Total	Illumina TruSeq Nano DNA HT library preparation kit	None	Male		NA	Macrophages	Immunosuppression
GSM3496512	GSE123141	Blood IBD	IBD	Blood	Total	Illumina TruSeq Nano DNA HT library preparation kit	None	Female		NA	Macrophages	None
GSM3496513	GSE123141	Blood IBD	IBD	Blood	Total	Illumina TruSeq Nano DNA HT library preparation kit	None	Female		NA	Macrophages	Immunosuppression
GSM3496514	GSE123141	Blood IBD	IBD	Blood	Total	Illumina TruSeq Nano DNA HT library preparation kit	None	Male		NA	Macrophages	Immunosuppression
GSM3496515	GSE123141	Blood IBD	IBD	Blood	Total	Illumina TruSeq Nano DNA HT library preparation kit	None	Female		NA	Macrophages	Immunosuppression
GSM3496516	GSE123141	Blood IBD	IBD	Blood	Total	Illumina TruSeq Nano DNA HT library preparation kit	None	Male		NA	Macrophages	None
GSM3496517	GSE123141	Blood IBD	IBD	Blood	Total	Illumina TruSeq Nano DNA HT library preparation kit	None	Female		NA	Macrophages	Immunosuppression
GSM3496518	GSE123141	Blood IBD	IBD	Blood	Total	Illumina TruSeq Nano DNA HT library preparation kit	None	Female		NA	Macrophages	Immunosuppression
GSM3496519	GSE123141	Blood IBD	IBD	Blood	Total	Illumina TruSeq Nano DNA HT library preparation kit	None	Male		NA	Macrophages	None
GSM3496520	GSE123141	Blood Control	Control	Blood	Total	Illumina TruSeq Nano DNA HT library preparation kit	None	Male		NA	Macrophages	None

GSM3496521	GSE123141	Blood Control	Control	Blood	Total	Illumina TruSeq Nano DNA HT library preparation kit	None	Female		NA	Macrophages	None
GSM3496522	GSE123141	Blood Control	Control	Blood	Total	Illumina TruSeq Nano DNA HT library preparation kit	None	Male		NA	Macrophages	None
GSM3496523	GSE123141	Blood Control	Control	Blood	Total	Illumina TruSeq Nano DNA HT library preparation kit	None	Male		NA	Macrophages	None
GSM3496524	GSE123141	Blood Control	Control	Blood	Total	Illumina TruSeq Nano DNA HT library preparation kit	None	Male		NA	Macrophages	None
GSM3496525	GSE123141	Blood Control	Control	Blood	Total	Illumina TruSeq Nano DNA HT library preparation kit	None	Male		NA	Macrophages	None
GSM3496526	GSE123141	Blood Control	Control	Blood	Total	Illumina TruSeq Nano DNA HT library preparation kit	None	Male		NA	Macrophages	None
GSM3496527	GSE123141	Blood Control	Control	Blood	Total	Illumina TruSeq Nano DNA HT library preparation kit	None	Female		NA	Macrophages	None
GSM3496528	GSE123141	Blood Control	Control	Blood	Total	Illumina TruSeq Nano DNA HT library preparation kit	None	Female		NA	Macrophages	None
GSM3496529	GSE123141	Blood IBD	IBD	Blood	Total	Illumina TruSeq Nano DNA HT library preparation kit	None	Female		NA	Macrophages	None
GSM3496530	GSE123141	Blood IBD	IBD	Blood	Total	Illumina TruSeq Nano DNA HT library preparation kit	None	Male		NA	Macrophages	Immunosuppression
GSM3496531	GSE123141	Blood IBD	IBD	Blood	Total	Illumina TruSeq Nano DNA HT library preparation kit	None	Female		NA	Macrophages	None
GSM3496532	GSE123141	Blood IBD	IBD	Blood	Total	Illumina TruSeq Nano DNA HT library preparation kit	None	Male		NA	Macrophages	Immunosuppression
GSM3496533	GSE123141	Blood IBD	IBD	Blood	Total	Illumina TruSeq Nano DNA HT library preparation kit	None	Female		NA	Macrophages	None
GSM3496534	GSE123141	Blood IBD	IBD	Blood	Total	Illumina TruSeq Nano DNA HT library preparation kit	None	Male		NA	Macrophages	None

GSM3496535	GSE123141	Blood IBD	IBD	Blood	Total	Illumina TruSeq Nano DNA HT library preparation kit	None	Male		NA	Macrophages	Immunosuppression
GSM3496536	GSE123141	Blood IBD	IBD	Blood	Total	Illumina TruSeq Nano DNA HT library preparation kit	None	Female		NA	Macrophages	None
GSM3496537	GSE123141	Blood IBD	IBD	Blood	Total	Illumina TruSeq Nano DNA HT library preparation kit	None	Male		NA	Macrophages	Immunosuppression
GSM3496538	GSE123141	Blood IBD	IBD	Blood	Total	Illumina TruSeq Nano DNA HT library preparation kit	None	Male		NA	Macrophages	None
GSM3890958	GSE132732	Blood Control	Control	Blood	polyA	Illumina TruSeq Stranded mRNA LT library preparation kit	None	NA		NA	Macrophages	IL4
GSM3890959	GSE132732	Blood Control	Control	Blood	polyA	Illumina TruSeq Stranded mRNA LT library preparation kit	None	NA		NA	Macrophages	NA
GSM3890960	GSE132732	Blood Control	Control	Blood	polyA	Illumina TruSeq Stranded mRNA LT library preparation kit	None	NA		NA	Macrophages	IL4
GSM3890961	GSE132732	Blood Control	Control	Blood	polyA	Illumina TruSeq Stranded mRNA LT library preparation kit	None	NA		NA	Macrophages	NA
GSM3890962	GSE132732	Blood Control	Control	Blood	polyA	Illumina TruSeq Stranded mRNA LT library preparation kit	None	NA		NA	Macrophages	IL4
GSM3890963	GSE132732	Blood Control	Control	Blood	polyA	Illumina TruSeq Stranded mRNA LT library preparation kit	None	NA		NA	Macrophages	NA
GSM3995429	GSE135223	Blood IBD	IBD	Blood	polyA	Illumina NEBnext Ultra II RNA Library Prep Kit	Ficoll	NA		NA	Macrophages	NA
GSM3995430	GSE135223	Blood IBD	IBD	Blood	polyA	Illumina NEBnext Ultra II RNA Library Prep Kit	Ficoll	NA		NA	Macrophages	NA
GSM3995431	GSE135223	Blood IBD	IBD	Blood	polyA	Illumina NEBnext Ultra II RNA Library Prep Kit	Ficoll	NA		NA	Macrophages	NA
GSM3995432	GSE135223	Blood IBD	IBD	Blood	polyA	Illumina NEBnext Ultra II RNA Library Prep Kit	Ficoll	NA		NA	Macrophages	NA
GSM3995433	GSE135223	Blood IBD	IBD	Blood	polyA	Illumina NEBnext Ultra II RNA Library Prep Kit	Ficoll	NA		NA	Macrophages	NA
GSM3995434	GSE135223	Blood Control	Control	Blood	polyA	Illumina NEBnext Ultra II RNA Library Prep Kit	Ficoll	NA		NA	Macrophages	NA

GSM3995435	GSE135223	Blood Control	Control	Blood	polyA	Illumina NEBnext Ultra II RNA Library Prep Kit	Ficoll	NA		NA	Macrophages	NA
GSM3995436	GSE135223	Blood Control	Control	Blood	polyA	Illumina NEBnext Ultra II RNA Library Prep Kit	Ficoll	NA		NA	Macrophages	NA
GSM3995437	GSE135223	Blood Control	Control	Blood	polyA	Illumina NEBnext Ultra II RNA Library Prep Kit	Ficoll	NA		NA	Macrophages	NA
GSM3995438	GSE135223	Blood Control	Control	Blood	polyA	Illumina NEBnext Ultra II RNA Library Prep Kit	Ficoll	NA		NA	Macrophages	NA
GSM3995439	GSE135223	Blood Control	Control	Blood	polyA	Illumina NEBnext Ultra II RNA Library Prep Kit	Ficoll	NA		NA	Macrophages	NA
GSM3995440	GSE135223	Blood Control	Control	Blood	polyA	Illumina NEBnext Ultra II RNA Library Prep Kit	Ficoll	NA		NA	Macrophages	NA
GSM3995441	GSE135223	Blood Control	Control	Blood	polyA	Illumina NEBnext Ultra II RNA Library Prep Kit	Ficoll	NA		NA	Macrophages	NA
GSM3995442	GSE135223	Blood IBD	IBD	Blood	polyA	Illumina NEBnext Ultra II RNA Library Prep Kit	Ficoll	NA		NA	Macrophages	NA
GSM3995443	GSE135223	Blood IBD	IBD	Blood	polyA	Illumina NEBnext Ultra II RNA Library Prep Kit	Ficoll	NA		NA	Macrophages	NA
GSM3995444	GSE135223	Blood IBD	IBD	Blood	polyA	Illumina NEBnext Ultra II RNA Library Prep Kit	Ficoll	NA		NA	Macrophages	NA
GSM3995445	GSE135223	Blood IBD	IBD	Blood	polyA	Illumina NEBnext Ultra II RNA Library Prep Kit	Ficoll	NA		NA	Macrophages	NA
GSM3995446	GSE135223	Blood IBD	IBD	Blood	polyA	Illumina NEBnext Ultra II RNA Library Prep Kit	Ficoll	NA		NA	Macrophages	NA
GSM4559002	GSE150805	Blood Control	Control	Blood	Total	NA	NA	NA		NA	T-cells	NA
GSM4559003	GSE150805	Blood Control	Control	Blood	Total	NA	NA	NA		NA	T-cells	IL17A
GSM4559004	GSE150805	Blood Control	Control	Blood	Total	NA	NA	NA		NA	T-cells	IL17F
GSM4559005	GSE150805	Blood Control	Control	Blood	Total	NA	NA	NA		NA	T-cells	ILL17A IL17F
GSM4559006	GSE150805	Blood Control	Control	Blood	Total	NA	NA	NA		NA	T-cells	NA
GSM4559007	GSE150805	Blood Control	Control	Blood	Total	NA	NA	NA		NA	T-cells	IL17A
GSM4559008	GSE150805	Blood Control	Control	Blood	Total	NA	NA	NA		NA	T-cells	IL17F
GSM4559009	GSE150805	Blood Control	Control	Blood	Total	NA	NA	NA		NA	T-cells	ILL17A IL17F
GSM4559010	GSE150805	Blood Control	Control	Blood	Total	NA	NA	NA		NA	T-cells	NA
GSM4559011	GSE150805	Blood Control	Control	Blood	Total	NA	NA	NA		NA	T-cells	IL17A
GSM4559012	GSE150805	Blood Control	Control	Blood	Total	NA	NA	NA		NA	T-cells	IL17F
GSM4559013	GSE150805	Blood Control	Control	Blood	Total	NA	NA	NA		NA	T-cells	ILL17A IL17F
GSM5088149	GSE166924	Blood Control	Control	Blood	polyA	Smart-seq2	FACS	NA		NA	Eosinophils	NA
GSM5088151	GSE166924	Blood Control	Control	Blood	polyA	Smart-seq2	FACS	NA		NA	Mononuclear phagocytes	NA
GSM5088153	GSE166924	Blood Control	Control	Blood	polyA	Smart-seq2	FACS	NA		NA	Neutrophils	NA
GSM5088155	GSE166924	Blood IBD	IBD	Blood	polyA	Smart-seq2	FACS	Female	27	NA	Eosinophils	NA
GSM5088157	GSE166924	Blood IBD	IBD	Blood	polyA	Smart-seq2	FACS	Female	27	NA	Mononuclear phagocytes	NA
GSM5088159	GSE166924	Blood IBD	IBD	Blood	polyA	Smart-seq2	FACS	Female	27	NA	Neutrophils	NA
GSM5088161	GSE166924	Blood IBD	IBD	Blood	polyA	Smart-seq2	FACS	Female	27	NA	Stromal cells	NA

GSM5088163	GSE166924	Blood IBD	IBD	Blood	polyA	Smart-seq2	FACS	Female	27	NA	Eosinophils	NA
GSM5088164	GSE166924	Blood IBD	IBD	Blood	polyA	Smart-seq2	FACS	Female	27	NA	Mononuclear phagocytes	NA
GSM5088166	GSE166924	Blood IBD	IBD	Blood	polyA	Smart-seq2	FACS	Female	27	NA	Neutrophils	NA
GSM5088168	GSE166924	Blood IBD	IBD	Blood	polyA	Smart-seq2	FACS	Female	27	NA	Stromal cells	NA
GSM5088170	GSE166924	Blood IBD	IBD	Blood	polyA	Smart-seq2	FACS	Male	31	NA	Eosinophils	NA
GSM5088172	GSE166924	Blood IBD	IBD	Blood	polyA	Smart-seq2	FACS	Male	31	NA	Mononuclear phagocytes	NA
GSM5088174	GSE166924	Blood IBD	IBD	Blood	polyA	Smart-seq2	FACS	Male	31	NA	Neutrophils	NA
GSM5088176	GSE166924	Blood IBD	IBD	Blood	polyA	Smart-seq2	FACS	Male	31	NA	Stromal cells	NA
GSM5088178	GSE166924	Blood IBD	IBD	Blood	polyA	Smart-seq2	FACS	Male	31	NA	Eosinophils	NA
GSM5088180	GSE166924	Blood IBD	IBD	Blood	polyA	Smart-seq2	FACS	Male	31	NA	Mononuclear phagocytes	NA
GSM5088182	GSE166924	Blood IBD	IBD	Blood	polyA	Smart-seq2	FACS	Male	31	NA	Neutrophils	NA
GSM5088184	GSE166924	Blood IBD	IBD	Blood	polyA	Smart-seq2	FACS	Male	31	NA	Stromal cells	NA
GSM5088186	GSE166924	Blood IBD	IBD	Blood	polyA	Smart-seq2	FACS	Female	49	NA	Eosinophils	NA
GSM5088188	GSE166924	Blood IBD	IBD	Blood	polyA	Smart-seq2	FACS	Female	49	NA	Mononuclear phagocytes	NA
GSM5088190	GSE166924	Blood IBD	IBD	Blood	polyA	Smart-seq2	FACS	Female	49	NA	Neutrophils	NA
GSM5088192	GSE166924	Blood IBD	IBD	Blood	polyA	Smart-seq2	FACS	Female	49	NA	Stromal cells	NA
GSM5088194	GSE166924	Blood IBD	IBD	Blood	polyA	Smart-seq2	FACS	Female	49	NA	Eosinophils	NA
GSM5088196	GSE166924	Blood IBD	IBD	Blood	polyA	Smart-seq2	FACS	Female	49	NA	Mononuclear phagocytes	NA
GSM5088198	GSE166924	Blood IBD	IBD	Blood	polyA	Smart-seq2	FACS	Female	49	NA	Neutrophils	NA
GSM5088200	GSE166924	Blood IBD	IBD	Blood	polyA	Smart-seq2	FACS	Female	49	NA	Stromal cells	NA
GSM5088202	GSE166924	Blood IBD	IBD	Blood	polyA	Smart-seq2	FACS	Male	50	NA	Eosinophils	NA
GSM5088204	GSE166924	Blood IBD	IBD	Blood	polyA	Smart-seq2	FACS	Male	50	NA	Mononuclear phagocytes	NA
GSM5088206	GSE166924	Blood IBD	IBD	Blood	polyA	Smart-seq2	FACS	Male	50	NA	Neutrophils	NA
GSM5088208	GSE166924	Blood IBD	IBD	Blood	polyA	Smart-seq2	FACS	Male	50	NA	Stromal cells	NA
GSM5088210	GSE166924	Blood IBD	IBD	Blood	polyA	Smart-seq2	FACS	Male	20	NA	Eosinophils	NA
GSM5088212	GSE166924	Blood IBD	IBD	Blood	polyA	Smart-seq2	FACS	Male	20	NA	Mononuclear phagocytes	NA
GSM5088214	GSE166924	Blood IBD	IBD	Blood	polyA	Smart-seq2	FACS	Male	20	NA	Neutrophils	NA
GSM5088216	GSE166924	Blood IBD	IBD	Blood	polyA	Smart-seq2	FACS	Male	20	NA	Stromal cells	NA
GSM5088218	GSE166924	Blood IBD	IBD	Blood	polyA	Smart-seq2	FACS	Male	66	NA	Eosinophils	NA
GSM5088220	GSE166924	Blood IBD	IBD	Blood	polyA	Smart-seq2	FACS	Male	66	NA	Mononuclear phagocytes	NA
GSM5088222	GSE166924	Blood IBD	IBD	Blood	polyA	Smart-seq2	FACS	Male	66	NA	Neutrophils	NA
GSM5088224	GSE166924	Blood IBD	IBD	Blood	polyA	Smart-seq2	FACS	Male	66	NA	Stromal cells	NA
GSM5088226	GSE166924	Blood IBD	IBD	Blood	polyA	Smart-seq2	FACS	Female	59	NA	Eosinophils	NA
GSM5088227	GSE166924	Blood IBD	IBD	Blood	polyA	Smart-seq2	FACS	Female	59	NA	Mononuclear phagocytes	NA
GSM5088229	GSE166924	Blood IBD	IBD	Blood	polyA	Smart-seq2	FACS	Female	59	NA	Neutrophils	NA
GSM5088231	GSE166924	Blood IBD	IBD	Blood	polyA	Smart-seq2	FACS	Female	59	NA	Stromal cells	NA
GSM5088233	GSE166924	Blood Control	Control	Blood	polyA	Smart-seq2	FACS	NA		NA	Eosinophils	NA
GSM5088235	GSE166924	Blood Control	Control	Blood	polyA	Smart-seq2	FACS	NA		NA	Mononuclear phagocytes	NA
GSM5088237	GSE166924	Blood Control	Control	Blood	polyA	Smart-seq2	FACS	NA		NA	Neutrophils	NA
GSM5088239	GSE166924	Blood IBD	IBD	Blood	polyA	Smart-seq2	FACS	Male	29	NA	Eosinophils	NA
GSM5088241	GSE166924	Blood IBD	IBD	Blood	polyA	Smart-seq2	FACS	Male	29	NA	Mononuclear phagocytes	NA
GSM5088243	GSE166924	Blood IBD	IBD	Blood	polyA	Smart-seq2	FACS	Male	29	NA	Neutrophils	NA
GSM5088245	GSE166924	Blood IBD	IBD	Blood	polyA	Smart-seq2	FACS	Male	29	NA	Stromal cells	NA
GSM5088247	GSE166924	Blood IBD	IBD	Blood	polyA	Smart-seq2	FACS	Male	29	NA	Eosinophils	NA

GSM5088249	GSE166924	Blood IBD	IBD	Blood	polyA	Smart-seq2	FACS	Male	29	NA	Mononuclear phagocytes	NA
GSM5088251	GSE166924	Blood IBD	IBD	Blood	polyA	Smart-seq2	FACS	Male	29	NA	Neutrophils	NA
GSM5088253	GSE166924	Blood IBD	IBD	Blood	polyA	Smart-seq2	FACS	Male	29	NA	Stromal cells	NA
GSM5088255	GSE166924	Blood IBD	IBD	Blood	polyA	Smart-seq2	FACS	Female	39	NA	Eosinophils	NA
GSM5088257	GSE166924	Blood IBD	IBD	Blood	polyA	Smart-seq2	FACS	Female	39	NA	Mononuclear phagocytes	NA
GSM5088259	GSE166924	Blood IBD	IBD	Blood	polyA	Smart-seq2	FACS	Female	39	NA	Neutrophils	NA
GSM5088261	GSE166924	Blood IBD	IBD	Blood	polyA	Smart-seq2	FACS	Female	39	NA	Stromal cells	NA
GSM5088263	GSE166924	Blood IBD	IBD	Blood	polyA	Smart-seq2	FACS	Male	35	NA	Eosinophils	NA
GSM5088265	GSE166924	Blood IBD	IBD	Blood	polyA	Smart-seq2	FACS	Male	35	NA	Mononuclear phagocytes	NA
GSM5088267	GSE166924	Blood IBD	IBD	Blood	polyA	Smart-seq2	FACS	Male	35	NA	Neutrophils	NA
GSM5088269	GSE166924	Blood IBD	IBD	Blood	polyA	Smart-seq2	FACS	Male	35	NA	Stromal cells	NA
GSM5088271	GSE166924	Blood IBD	IBD	Blood	polyA	Smart-seq2	FACS	Female	72	NA	Eosinophils	NA
GSM5088273	GSE166924	Blood IBD	IBD	Blood	polyA	Smart-seq2	FACS	Female	72	NA	Mononuclear phagocytes	NA
GSM5088275	GSE166924	Blood IBD	IBD	Blood	polyA	Smart-seq2	FACS	Female	72	NA	Neutrophils	NA
GSM5088277	GSE166924	Blood IBD	IBD	Blood	polyA	Smart-seq2	FACS	Female	72	NA	Stromal cells	NA
GSM5088279	GSE166924	Blood IBD	IBD	Blood	polyA	Smart-seq2	FACS	Female	27	NA	Eosinophils	NA
GSM5088281	GSE166924	Blood IBD	IBD	Blood	polyA	Smart-seq2	FACS	Female	27	NA	Mononuclear phagocytes	NA
GSM5088283	GSE166924	Blood IBD	IBD	Blood	polyA	Smart-seq2	FACS	Female	27	NA	Neutrophils	NA
GSM5088285	GSE166924	Blood IBD	IBD	Blood	polyA	Smart-seq2	FACS	Female	27	NA	Stromal cells	NA
GSM5088287	GSE166924	Blood IBD	IBD	Blood	polyA	Smart-seq2	FACS	Male	22	NA	Eosinophils	NA
GSM5088289	GSE166924	Blood IBD	IBD	Blood	polyA	Smart-seq2	FACS	Male	22	NA	Mononuclear phagocytes	NA
GSM5088291	GSE166924	Blood IBD	IBD	Blood	polyA	Smart-seq2	FACS	Male	22	NA	Neutrophils	NA
GSM5088293	GSE166924	Blood IBD	IBD	Blood	polyA	Smart-seq2	FACS	Male	22	NA	Stromal cells	NA
GSM5088295	GSE166924	Blood Control	Control	Blood	polyA	Smart-seq2	FACS	NA		NA	Eosinophils	NA
GSM5088297	GSE166924	Blood Control	Control	Blood	polyA	Smart-seq2	FACS	NA		NA	Mononuclear phagocytes	NA
GSM5088299	GSE166924	Blood Control	Control	Blood	polyA	Smart-seq2	FACS	NA		NA	Neutrophils	NA
GSM5088300	GSE166924	Blood Control	Control	Blood	polyA	Smart-seq2	FACS	NA		NA	Eosinophils	NA
GSM5088302	GSE166924	Blood Control	Control	Blood	polyA	Smart-seq2	FACS	NA		NA	Mononuclear phagocytes	NA
GSM5088304	GSE166924	Blood Control	Control	Blood	polyA	Smart-seq2	FACS	NA		NA	Neutrophils	NA
GSM5088306	GSE166924	Blood Control	Control	Blood	polyA	Smart-seq2	FACS	NA		NA	Eosinophils	NA
GSM5088308	GSE166924	Blood Control	Control	Blood	polyA	Smart-seq2	FACS	NA		NA	Mononuclear phagocytes	NA
GSM5088310	GSE166924	Blood Control	Control	Blood	polyA	Smart-seq2	FACS	NA		NA	Neutrophils	NA
GSM5088312	GSE166924	Blood IBD	IBD	Blood	polyA	Smart-seq2	FACS	Male	36	NA	Eosinophils	NA
GSM5088314	GSE166924	Blood IBD	IBD	Blood	polyA	Smart-seq2	FACS	Male	36	NA	Mononuclear phagocytes	NA
GSM5088315	GSE166924	Blood IBD	IBD	Blood	polyA	Smart-seq2	FACS	Male	36	NA	Neutrophils	NA
GSM5088317	GSE166924	Blood IBD	IBD	Blood	polyA	Smart-seq2	FACS	Female	39	NA	Eosinophils	NA
GSM5088319	GSE166924	Blood IBD	IBD	Blood	polyA	Smart-seq2	FACS	Female	39	NA	Mononuclear phagocytes	NA
GSM5088321	GSE166924	Blood IBD	IBD	Blood	polyA	Smart-seq2	FACS	Female	39	NA	Neutrophils	NA
GSM5088323	GSE166924	Blood IBD	IBD	Blood	polyA	Smart-seq2	FACS	Female	23	NA	Eosinophils	NA
GSM5088325	GSE166924	Blood IBD	IBD	Blood	polyA	Smart-seq2	FACS	Female	23	NA	Mononuclear phagocytes	NA
GSM5088327	GSE166924	Blood IBD	IBD	Blood	polyA	Smart-seq2	FACS	Female	23	NA	Neutrophils	NA
GSM5088329	GSE166924	Blood IBD	IBD	Blood	polyA	Smart-seq2	FACS	Male	82	NA	Eosinophils	NA
GSM5088330	GSE166924	Blood IBD	IBD	Blood	polyA	Smart-seq2	FACS	Male	82	NA	Mononuclear phagocytes	NA

GSM5088332	GSE166924	Blood IBD	IBD	Blood	polyA	Smart-seq2	FACS	Male	82	NA	Neutrophils	NA
